# The Ras small GTPase RSR1 regulates cellulase production in *Trichoderma reesei*

**DOI:** 10.1186/s13068-023-02341-z

**Published:** 2023-05-23

**Authors:** Ni Li, Zhouyuan Qiu, Wanchuan Cai, Yaling Shen, Dongzhi Wei, Yumeng Chen, Wei Wang

**Affiliations:** 1 The State Key Laboratory of Bioreactor Engineering, East China University of Science and Technology, 130 Meilong Road, P.O.B. 311, Shanghai, 200237 China; 2 Jiangsu Yiming Biological Technology Co., Ltd., Suqian, 223699 Jiangsu China

**Keywords:** Ras small GTPase RSR1, Cyclic AMP, PKA, Filamentous fungi, *Trichoderma reesei*, Cellulase, Signaling

## Abstract

**Background:**

Lignocellulose is the most abundant renewable resource in the world and has attracted widespread attention. It can be hydrolyzed into sugars with the help of cellulases and hemicellulases that are secreted by filamentous fungi. Several studies have revealed that the Ras small GTPase superfamily regulates important cellular physiological processes, including synthesis of metabolites, sporulation, and cell growth and differentiation. However, it remains unknown how and to what extent Ras small GTPases participate in cellulase production.

**Results:**

In this study, we found that the putative Ras small GTPase RSR1 negatively regulated the expression of cellulases and xylanases. Deletion of *rsr1* (∆*rsr1*) significantly increased cellulase production and decreased the expression levels of ACY1-cAMP-protein kinase A (PKA) signaling pathway genes and the concentration of intracellular cyclic adenosine monophosphate (cAMP). Loss of *acy1* based on ∆*rsr1* (∆*rsr1*∆*acy1*) could further increase cellulase production and the expression levels of cellulase genes, while overexpression of *acy1* based on ∆*rsr1* (∆*rsr1*-OE*acy1*) significantly reduced cellulase production and transcriptional levels of cellulase genes. In addition, our results revealed that RSR1 negatively controlled cellulase production via the ACY1-cAMP-PKA pathway. Transcriptome analysis revealed significantly increased expression of three G-protein coupled receptors (GPCRs; *tre62462*, *tre58767*, and *tre53238*) and approximately two-fold higher expression of ACE3 and XYR1, which transcriptionally activated cellulases with the loss of *rsr1*. ∆*rsr1*∆ *tre62462* exhibited a decrease in cellulase activity compared to ∆*rsr1*, while that of ∆*rsr1*∆*tre58767* and ∆*rsr1*∆*tre53238* showed a remarkable improvement compared to ∆*rsr1.* These findings revealed that GPCRs on the membrane may sense extracellular signals and transmit them to *rsr1* and then to ACY1-cAMP-PKA, thereby negatively controlling the expression of the cellulase activators ACE3 and XYR1. These data indicate the crucial role of Ras small GTPases in regulating cellulase gene expression.

**Conclusions:**

Here, we demonstrate that some GPCRs and Ras small GTPases play key roles in the regulation of cellulase genes in *Trichoderma reesei*. Understanding the roles of these components in the regulation of cellulase gene transcription and the signaling processes in *T. reesei* can lay the groundwork for understanding and transforming other filamentous fungi.

**Supplementary Information:**

The online version contains supplementary material available at 10.1186/s13068-023-02341-z.

## Background

*Trichoderma reesei* abundantly produces extracellular cellulases and hemicellulases, which catalyze the degradation of lignocellulose to monosaccharides [1–3]. This fungus is a primary source of enzymes for the production of bioethanol [4–8]. A major issue associated with the cost-effective production of bioethanol is optimization of the fungal platform to increase enzyme production [9, 10]. Many transcription factors play a vital role in the process of cellulase production [11], including the main repressor, CRE1, which is related to carbon catabolite repression (CCR) [12–14], and the crucial transcription activators XYR1 [15–17] and ACE3 [18–20]. Notably, *T. reesei* only produces enzymes abundantly in the presence of inducing carbon sources, such as Avicel and lactose [21]. The process of cellulase synthesis, stimulated by extracellular signals, is very complex [10]. Understanding the signal-sensing pathways in *T. reesei* will have a major impact on enzyme preparation and fuel production.

protein-coupled receptors (GPCRs) receive extracellular signals and may transmit them through Ras small GTPases [22–24], which in turn, transmit them downstream to various effectors. Ras small GTPases are binary molecular switches between the active [guanosine triphosphate (GTP) binding] and inactive [guanosine diphosphate (GDP) binding] conformations [25–27]. The activity of Ras small GTPases is controlled by two proteins: guanine nucleotide exchange factors (GEFs) and GTPase activation proteins (GAPs) [28]. When combined with GDP, the switch is closed, and an inactive structure exists [29]. However, Ras GEFs control the exchange of GDP with GTP, switching to the activated state [30]. The main functions of Ras GAPs are to hydrolyze GTP bound to Ras small GTPases to GDP and promote the inactivity of Ras small GTPases. In *Saccharomyces cerevisiae* and *Candida albicans*, activated Ras small GTPases transmit signals to the downstream signal-receiving kinase adenylate cyclase 1 (ACY1), which in turn, transmits them to protein kinase A (PKA) via cyclic adenosine monophosphate (cAMP), a vital second messenger [31, 32]. ACY1-cAMP-PKA is an important pathway that affects metabolism, enzyme production [12, 33], sporulation [33], vegetative growth [34, 35], and iron homeostasis [36].

In *T. reesei*, two Ras small GTPase genes, *TrRas1* and *TrRas2*, are important for numerous cellular processes, such as hyphae branching, sporulation, and cAMP level adjustment [37]. In *S. cerevisiae*, *Ras1* and *Ras2* homologs regulate cell cycle progression through the cAMP signaling pathway to sense extracellular glucose signals [38, 39]. Additionally, *S. cerevisiae Ras2* controls pseudohyphal differentiation in cooperation with the cAMP pathways [38]. In the human pathogen, *C. albicans*, *Ras1* homologs control cellular morphology and virulence through the cAMP-PKA signaling pathway [40]. Further, *Ras1* reportedly controls filamentation, mating, and growth at elevated temperatures, which is necessary for virulence in *Cryptococcus neoformans* via the cAMP-dependent signaling cascade [41]. In *Fusarium graminearum*, Ras GEF FgCdc25 regulates virulence and fungal development via the cAMP signaling pathway [25]. Previous studies have revealed that the Ras small GTPase superfamily regulates important cellular physiological processes [37–41], including the synthesis of metabolites, sporulation, cell growth, and cell differentiation. However, there is a lack of research on the Ras small GTPase superfamily and their connection to the ACY1-cAMP-PKA pathway in *T. reesei*, which sparked our research interest.

In the present study, five Ras GTPases, four Ras GEFs, and two Ras GAPs from *T. reesei* [42] were knocked out to investigate the role of Ras small GTPases in cellulose-induced signal transduction and cellulase production. Intracellular cAMP concentration, expression of ACY1-cAMP-PKA pathway genes, and comparative transcriptome data were analyzed in the parent and deletion strain. This study provides new perspectives for improving cellulase production and insights into the mechanism of cellulase regulation by Ras small GTPases in *T. reesei*.

## Results

### Ras small GTPase RSR1 negatively regulates cellulase and xylanase production

Using the BLAST online website in NCBI, 11 putative Ras subfamily genes were found in the genome of *T. reesei* (Table 1), including five Ras GTPases, four Ras GEFs, and two Ras GAPs. We constructed 11 putative knockout strains (Additional file 1: Table S1) by homologous recombination in the wild-type strain QM6a to explore the effect of these genes on cellulase production. Two of the Ras GTPases (*tre107035* and *tre66480*) and one Ras GEF (*tre70548*) could not be knocked out. These three genes may play a decisive role in growth, leading to death by gene knockout. The loss of another three genes had a significant effect on enzyme activity (Table 1). Deletion of the *TrRas2* gene led to a 60 ± 4.58% decrease in cellulase activity, which is consistent with the results obtained by Zhang et al. [37]. The loss of *tre107369* slightly increased cellulase activity by 10 ± 1.04% compared to the wild-type strain. The *rsr1* deletion mutant (Δ*rsr1* strain) displayed significantly increased (100 ± 8.12%) cellulase activity compared to the wild-type strain QM6a in the presence of 1% (w/v) Avicel.

**Table 1:** Effect of the deletion of 11 putative Ras subfamily genes on cellulase production

Family	Classification	Gene ID	State	Change	Multiple
Ras	Ras GTPase	*TrRas1*	√	No change	No
		*TrRas2*	√	Down	60 ± 4.58%
		*rsr1*	√	Up	100 ± 8.12%
		*tre107035*	×	Unknown	Unknown
		*tre66480*	×	Unknown	Unknown
	Ras GEF	*tre34726*	√	No change	No
		*tre107369*	√	Up	10 ± 1.04%
		*tre67275*	√	No change	No
		*tre70548*	×	Unknown	Unknown
	Ras GAP	*tre61408*	√	No change	No
		*tre81785*	√	No change	No

×  unsuccessful deletion, √ successful deletion

**Fig. 1:**
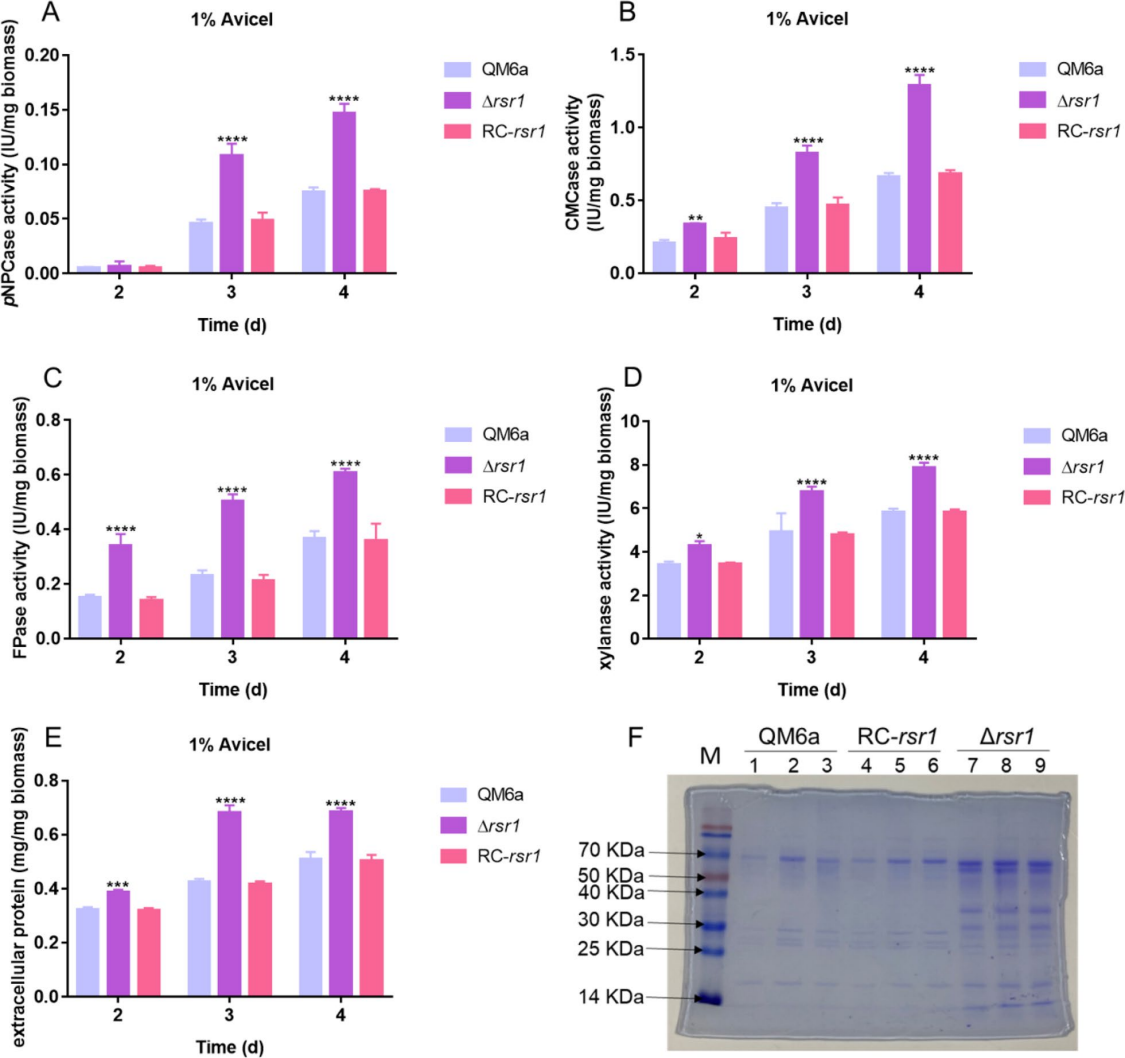
*rsr1* negatively regulates cellulase production. **A**–**E** Enzyme activities, including *p*NPCase (**A**), CMCase (**B**), FPase (**C**), and xylanase activity (**D**), and extracellular protein concentration (**E**) in *Trichoderma reesei* strains. (**F**) Sodium dodecyl-sulfate polyacrylamide gel electrophoresis (SDS-PAGE) analysis of the secretomes in supernatants of *T. reesei* strains. The measurements were made during culture with 1% (w/v) Avicel as the sole carbon source. Values are represented as mean ± standard deviation (SD) of the results from three independent experiments. Asterisks (*) indicate significant differences compared to the parental strain (Student’s *t*-test, *P < 0.05; **P < 0.01; ***P < 0.001; ****P < 0.0001)

**Fig. 2:**
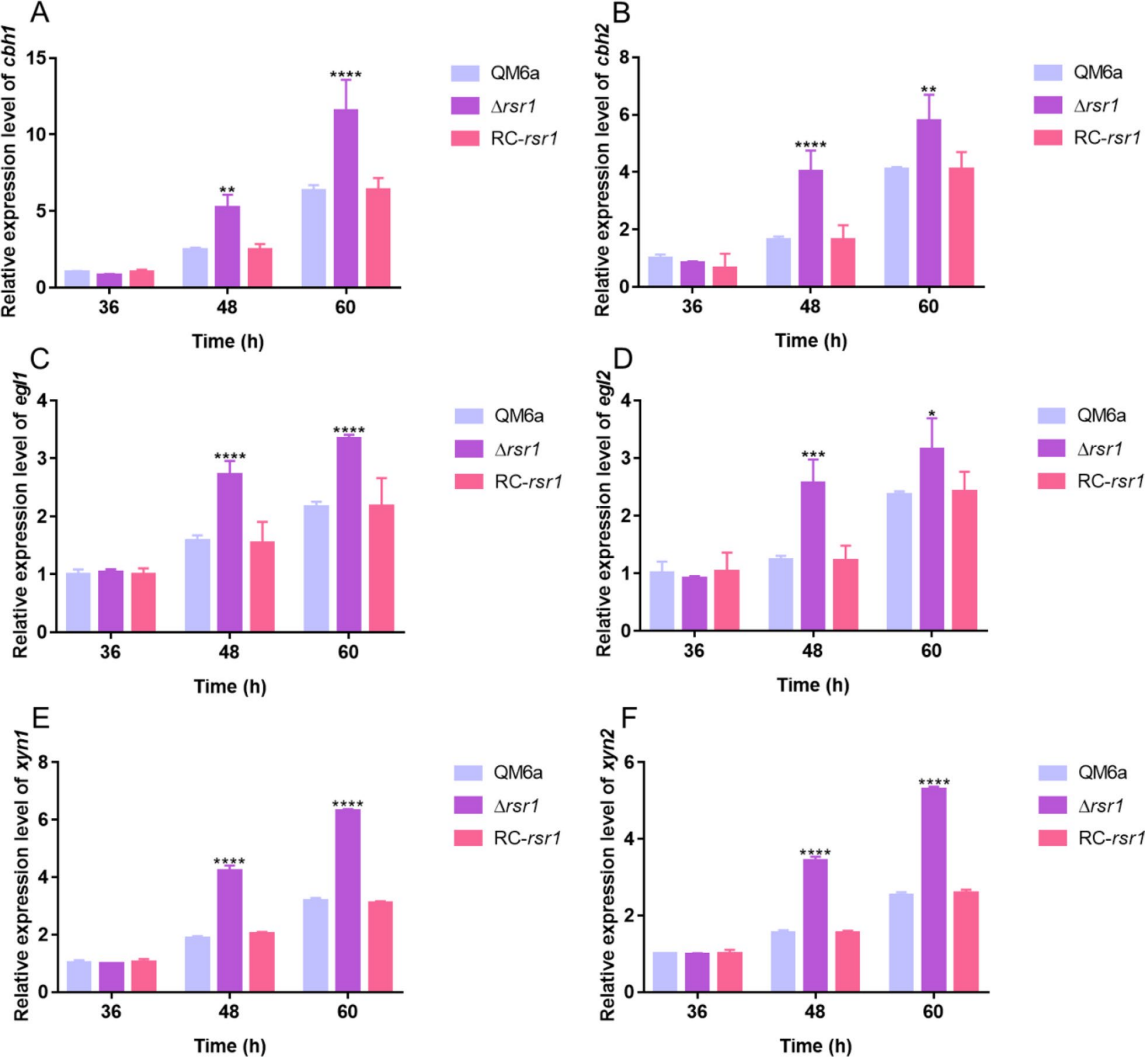
Expression levels of major cellulase genes when Avicel is used as the carbon source. Quantitative real-time polymerase chain reaction (RT-qPCR) was used to detect the transcriptional levels of four major cellulase genes, *cbh1* (**A**), *cbh2* (**B**), *egl1* (**C**), and *egl2* (**D**), and two major xylanase genes *xyn1* (**E**) and *xyn2* (**F**). Conidia of *T. reesei* were inoculated into MA liquid medium with 1% (w/v) Avicel as the sole carbon source. Sampling was done at 36, 48, and 60 h. The data are normalized to expression of QM6a at 36 h for each tested gene, with the *sar1* gene used as an endogenous control in all samples. Values are represented as mean ± SD of the results from three independent experiments. Asterisks (*) indicate significant differences compared to the parental strain (Student’s *t*-test, *P < 0.05; **P < 0.01; ***P < 0.001; ****P < 0.0001)

As shown in Additional file 2: Fig S1, when 2% (w/v) glucose was used as the sole carbon source, QM6a and Δ*rsr1* did not produce cellulases. Cellobiohydrolase (*p*NPCase), endoglucanase (CMCase), filter paper enzyme (FPase), and xylanase activities, and extracellular protein concentration were significantly improved in Δ*rsr1* compared to the wild-type strain QM6a in the presence of 1% (w/v) Avicel as the carbon source. The *p*NPCase, CMCase, and FPase activities of Δ*rsr1* were increased by approximately 100% compared with that of wild-type strain QM6a after 3–4 day of cultivation (Fig. 1A–C). The xylanase activity of Δ*rsr1* increased by 25–38% compared with that of QM6a (Fig. 1D). The extracellular protein concentration of the Δ*rsr1* strain was 34–60% higher than that of QM6a after 2 days of cultivation (Fig. 1E). We found that the deletion of *rsr1* had almost no effect on the growth of *T. reesei* (Additional file 3: Fig S2), indicating that the enhancement of cellulase production was not achieved by controlling biomass production. Additionally, we constructed a complementation strain (RC-*rsr1*) based on the deletion strain Δ*rsr1* (Additional file 4: Fig S3). The enzyme activities in RC-*rsr1* decreased to the same level as that in the wild-type strain QM6a (Fig. 1A–E). The results of the sodium dodecyl-sulfate polyacrylamide gel electrophoresis (SDS-PAGE) analysis (Fig. 1F) of the secretomes in supernatants of QM6a, RC-*rsr1*, and Δ*rsr1* were consistent with the cellulase activity data mentioned above. To investigate the effect of *rsr1* deletion on cellulase expression, we used real-time fluorescent quantitative polymerase chain reaction (RT-qPCR) to detect the transcriptional levels of the major cellulase and xylanase genes in the QM6a and Δ*rsr1* strains. As shown in Fig. 2A–F, deletion of *rsr1* significantly increased the expression of four major cellulase-encoding genes (*cbh1*, *cbh2*, *egl1*, and *egl2*) and two major xylanase genes (*xyn1* and *xyn2*). These results are consistent with the increased enzyme activity and extracellular protein production in the Δ*rsr1* strain (Fig. 1A–F). The results indicated that *rsr1* acts as a negative regulator of cellulase, xylanase, and secreted protein production.

**Fig. 3:**
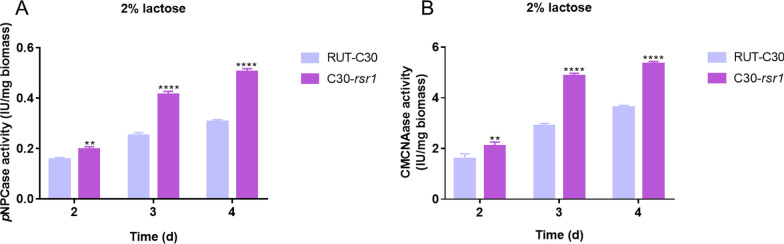
Effects of *rsr1* deletion on cellulase production in the *T. reesei* industrial strain, RUT-C30. The activities of *p*NPCase (**A**) and CMCase (**B**) of parental strain RUT-C30 and *rsr1* deletion strain C30-*rsr1* after culture in liquid 2 × Mandels’ medium containing 2% lactose. Values are the means ± SD of the results from three independent experiments. Asterisks (*) indicate a significant difference (Student’s *t*-test, **P < 0.01; ****P < 0.0001)

To study whether *rsr1* deletion can enhance cellulase production in the cellulase hyper-producer *T. reesei* RUT-C30, an industrial mutant [21], we also constructed the *rsr1* deletion strain C30-*rsr1* from RUT-C30 using the same method. Consistent with previous results, the loss of *rsr1* in RUT-C30 resulted in no significant differences in hyphal growth (Additional file 3: Fig S2). The *p*NPCase activity of C30- *rsr1* increased by > 70% (Fig. 3A) and CMCase activity increased by > 50% (Fig. 3B) compared with that of the parental strain RUT-C30 after cultivation for 3–4 days. These results suggested that *rsr1* also has a negative effect on cellulase production in the *T. reesei* mutant, RUT-C30, the deletion of which can effectively improve cellulase production. The data indicated that the *rsr1* deletion strain could possibly be used as a suitable candidate for high-yield cellulase production.

### Structure and phylogenetic analyses of RSR1

**Fig. 4:**
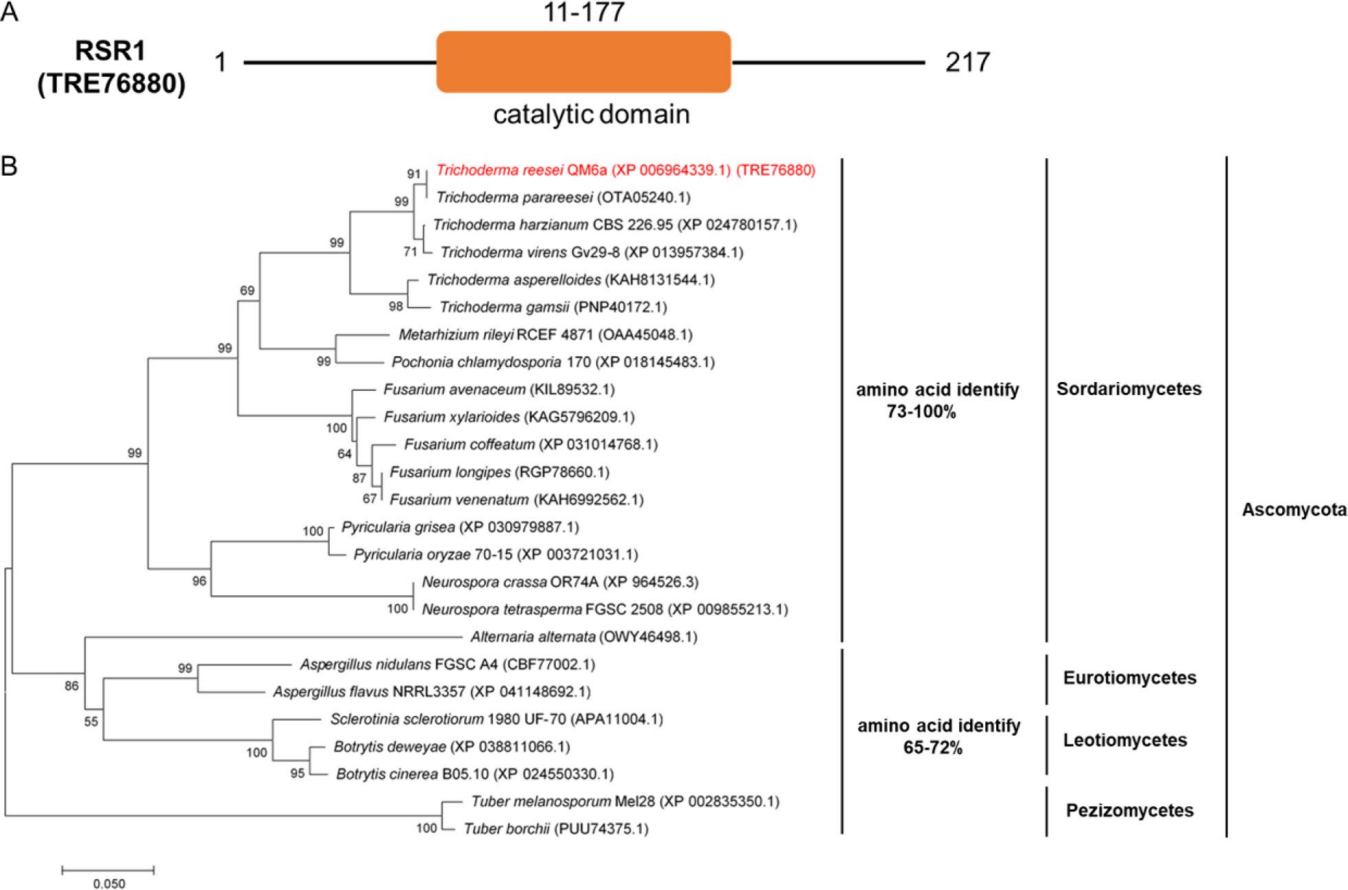
Bioinformatics analysis of the RSR1 gene. **A** The Pfam database was used to predict the functional domain of RSR1(TRE76880). A catalytic domain at position 11–177 (http://pfam.xfam.org) was identified. **B** Phylogenetic analyses of RSR1 protein and its orthologs. BLASTP of NCBI was used to find representative RSR1 orthologs. Then, amino acid sequence alignment and phylogenetic analysis was performed with ClustalW and the maximum likelihood tree was generated using MEGA7. The bootstrap supports calculated per 1,000 bootstrap replicates are represented by numbers on the tree branches

The RSR1 domain was predicted using the Pfam database (http://pfam.xfam.org). Only one GTPase catalytic domain was found at position 11–177 (Fig. 4A). Phylogenetic tree analysis (Fig. 4B) showed that RSR1 universally existed in some Ascomycota, including *Sordariomycetes*, *Eurotiomycetes*, *Leotiomycetes*, and *Pezizomycetes*, with amino acid similarity > 65%. The amino acid similarity of RSR1 homologs in *Trichoderma* exceeded 80%, and that in *Fusarium* and *Neurospora* ranged from 73 to 85%. *Neurospora crassa* is also an important filamentous fungus that breaks down lignocellulosic biomass and produces soluble sugars. The amino acid similarity of RSR1 homologs in this species was up to 85%. Although RSR1 is widely found in fungi, none of these putative RSR1 homologs were reported to regulate cellulase gene expression, which is worth studying.

### RSR1 negatively regulates the ACY1-cAMP-PKA pathway

**Fig. 5:**
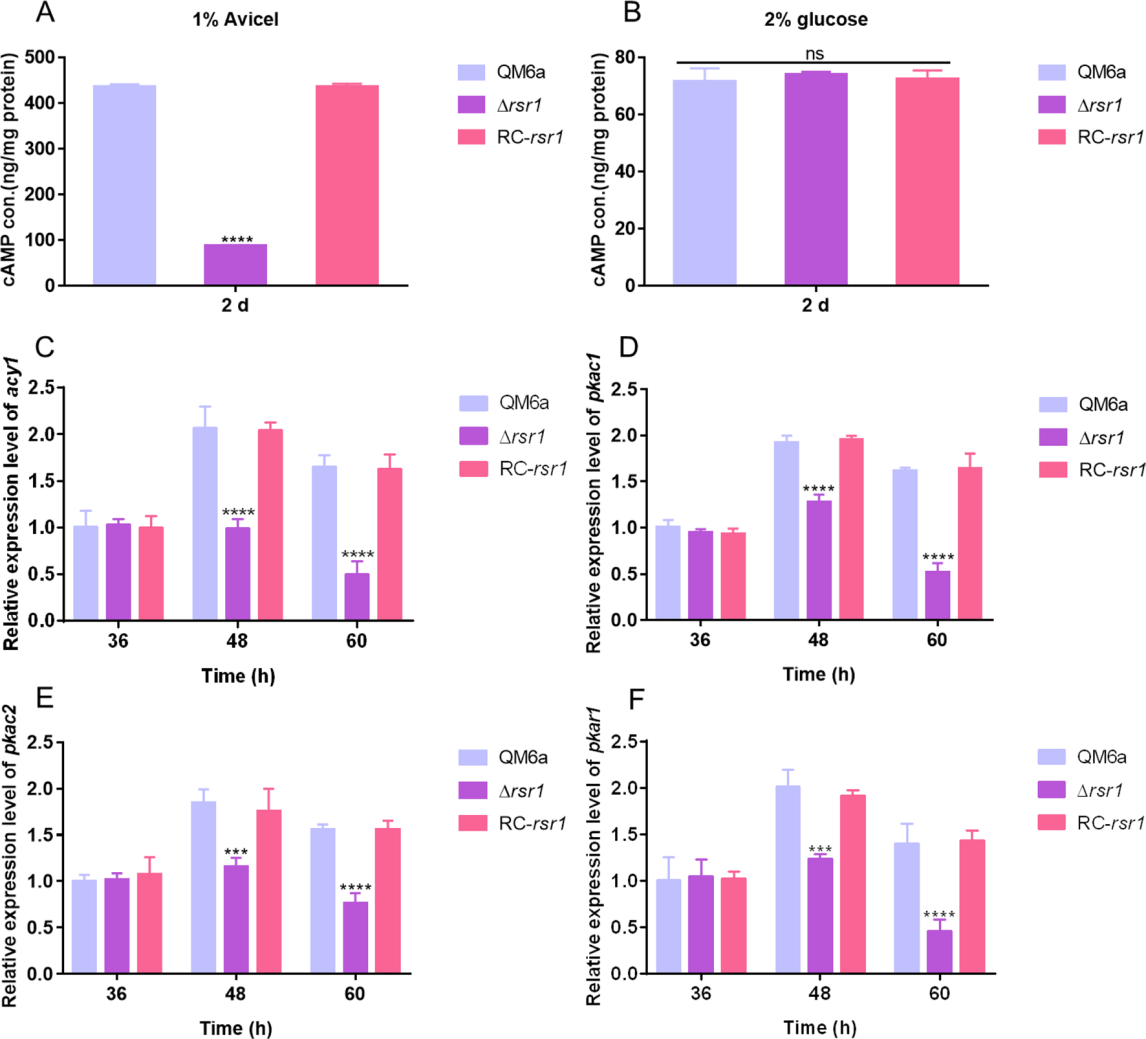
Effect of the loss of *rsr1* on the ACY1-cAMP-PKA pathway. **A**, **B** Intracellular cyclic adenosine monophosphate (cAMP) content of the original strain (QM6a), deletion strain (Δ*rsr1*), and complementation strain (RC-*rsr1*) were measured when 1% (w/v) Avicel (**A**) and 2% glucose (**B**) were the sole carbon source, respectively. Fresh conidia were generated from hyphae in MA liquid medium and intracellular cAMP content was measured at 2 days, as described in the Methods. (**C**–**F**) Transcriptional levels of *acy1* (**C**), *pkac1* (**D**), *pkac2* (**E**), and *pkar1* (**F**) detected by RT-qPCR in QM6a, Δ*rsr1*, and RC-*rsr1* strains with 1% (w/v) Avicel as the sole carbon source. Values are represented as mean ± SD of the results from three independent experiments. Asterisks (*) indicate significant differences compared to the parental strain (Student’s *t*-test, ***P < 0.001; ****P < 0.0001; ns represents no significant difference)

Ras small GTPase often transmits signals through the ACY1-cAMP-PKA signaling pathway [37, 40, 43–45]. Therefore, we detected the intracellular cAMP concentrations in the QM6a and Δ*rsr1* strains (Fig. 5A). The intracellular cAMP concentration in Δ*rsr1* was significantly decreased by 79.8% after 2 d of cultivation in the presence of 1% (w/v) Avicel compared to that observed in the original strain. The intracellular cAMP content in the complementation strain (RC-*rsr1*) was consistent with that of the original strain (Fig. 5A). However, the intracellular cAMP concentration in the Δ*rsr1* strain was unchanged compared with that in the wild-type and complementation strains, when glucose was used as the carbon source (Fig. 5B).

We also evaluated the transcription levels of the genes in the ACY1-cAMP-PKA signaling pathway in the QM6a and Δ*rsr1* strains. The transcription levels of the *acy1*, *pkac1*, *pkac2*, and *pkar1* genes in the knockout strain were remarkably reduced compared to the wild-type and complementation strains in the presence of 1% (w/v) Avicel (Fig. 5C–F). The expression levels of ACY1-cAMP-PKA genes in RC-*rsr1* recovered to the same levels as those in QM6a. However, their levels stayed consistent in Δ*rsr1*, QM6a, and RC-*rsr1* when glucose was used as the carbon source (data not shown). These results indicate that the deletion of *rsr1* results in downregulated expression levels of *acy1*, *pkac1*, *pkac2*, and *pkar1* genes and cAMP content at cellulase inducing conditions.

### RSR1 negatively regulates cellulase production through the ACY1-cAMP-PKA pathway

**Fig. 6:**
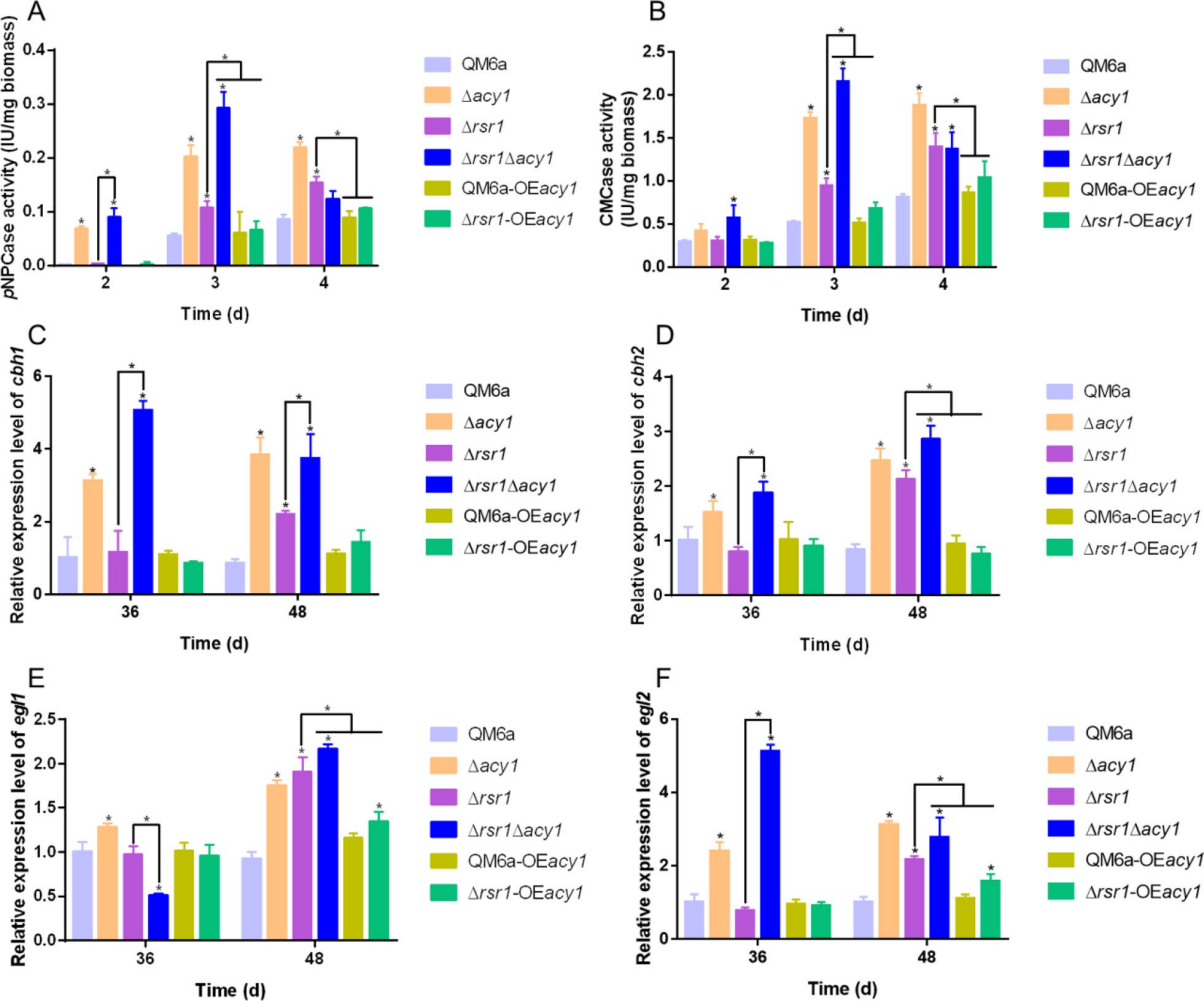
Effect of ACY1-cAMP-PKA pathway on *rsr1*-mediated cellulase overexpression. *p*NPCase activity (**A**) and CMCase activity (**B**) of *T. reesei* QM6a, Δ*acy1*, Δ*rsr1*, Δ*rsr1*Δ*acy1*, QM6a-OE*acy1* and Δ*rsr1*-OE*acy1* cultured in MA with 1% (w/v) Avicel at 2, 3, and 4 days. The transcript levels of *cbh1* (**C**), *cbh2* (**D**), *egl1* (**E**), and *egl2* (**F**) in *T. reesei* QM6a, Δ*acy1*, Δ*rsr1*, Δ*rsr1*Δ*acy1*, QM6a*-*OE*acy1* and Δ*rsr1-*OE*acy1* strains cultured in MA with 1% (w/v) Avicel at 36 and 48 h. Values are represented as mean ± SD of the results from three independent experiments. Asterisks (*) indicate significant differences compared to the parental strain (Student’s *t*-test, *P < 0.05)

To address if the decreased *acy1* transcriptional level and intracellular cAMP content mediates cellulase enhancement in the *rsr1* deletion strain, the deletion and overexpression of *acy1* were constructed in QM6a and Δ*rsr1*, and named *T. reesei* Δ*acy1*, Δ*rsr1*Δ*acy1*, QM6a-OE*acy1* and Δ*rsr1*-OE*acy1*, respectively (Additional file 4: Fig S3). As shown in Fig. 6A–B, the further improvement of *p*NPCase and CMCase activities was observed in Δ*rsr1*Δ*acy1* compared to Δ*rsr1* and QM6a. We found that the loss of *acy1* remarkably stimulated* p*NPCase and CMCase activities after 3 days of fermentation in the Δ*rsr1*Δ*acy1* strain, with an increase of approximately 174% and 128% compared with Δ*rsr1* and approximately 4.3- and 3.12-fold increase compared with QM6a, respectively. The enhanced cellulase production in the *rsr1* deletion strain was partly attenuated by the overexpression of *acy1* (Fig. 6A, B). The loss of *acy1* in the wild-type strain led to a significant improvement in *p*NPCase and CMCase activities (Fig. 6A, B), which also implied that *acy1* acted as a negative regulator of cellulase expression. Δ*rsr1*Δ*acy1* showed a higher level of cellulase production capacity compared to Δ*acy1* (Fig. 6A, B). The results indicated that *acy1* expression and intracellular cAMP concentration are involved in the negative regulation of cellulase production by RSR1.

To gain insight into how *acy1* influences the *rsr1*-mediated cellulase high-yield process at the transcriptional level, RT-qPCR analysis was performed to detect the expression levels of four main cellulase-encoding genes (*cbh1*, *cbh2*, *egl1*, and *egl2*) in the QM6a, Δ*acy1*, Δ*rsr1*, Δ*rsr1*Δ*acy1*, QM6a-OE*acy1*, and Δ*rsr1*-OE*acy1* strains. As shown in Fig. 6C–F, and consistent with previous data regarding *p*NPCase and CMCase activities (Fig. 6A, B), the deletion of *acy1* further enhanced the expression of cellulase-encoding genes in Δ*rsr1*Δ*acy1*, compared with that in Δ*rsr1*, while the overexpression of *acy1* partly abrogated the overexpression of cellulase-encoding genes in Δ*rsr1*-OE*acy1*, compared with that in Δ*rsr1*. These results indicated that the ACY1-cAMP-PKA pathway is required for *rsr1* to negatively regulate cellulase production. For the first time, we proved that ACY1 acts as a negative regulator of cellulase in the RSR1 signal transduction pathway.

### Transcriptional profile of the Δrsr1 mutant cultured in the presence of Avicel

**Fig. 7:**
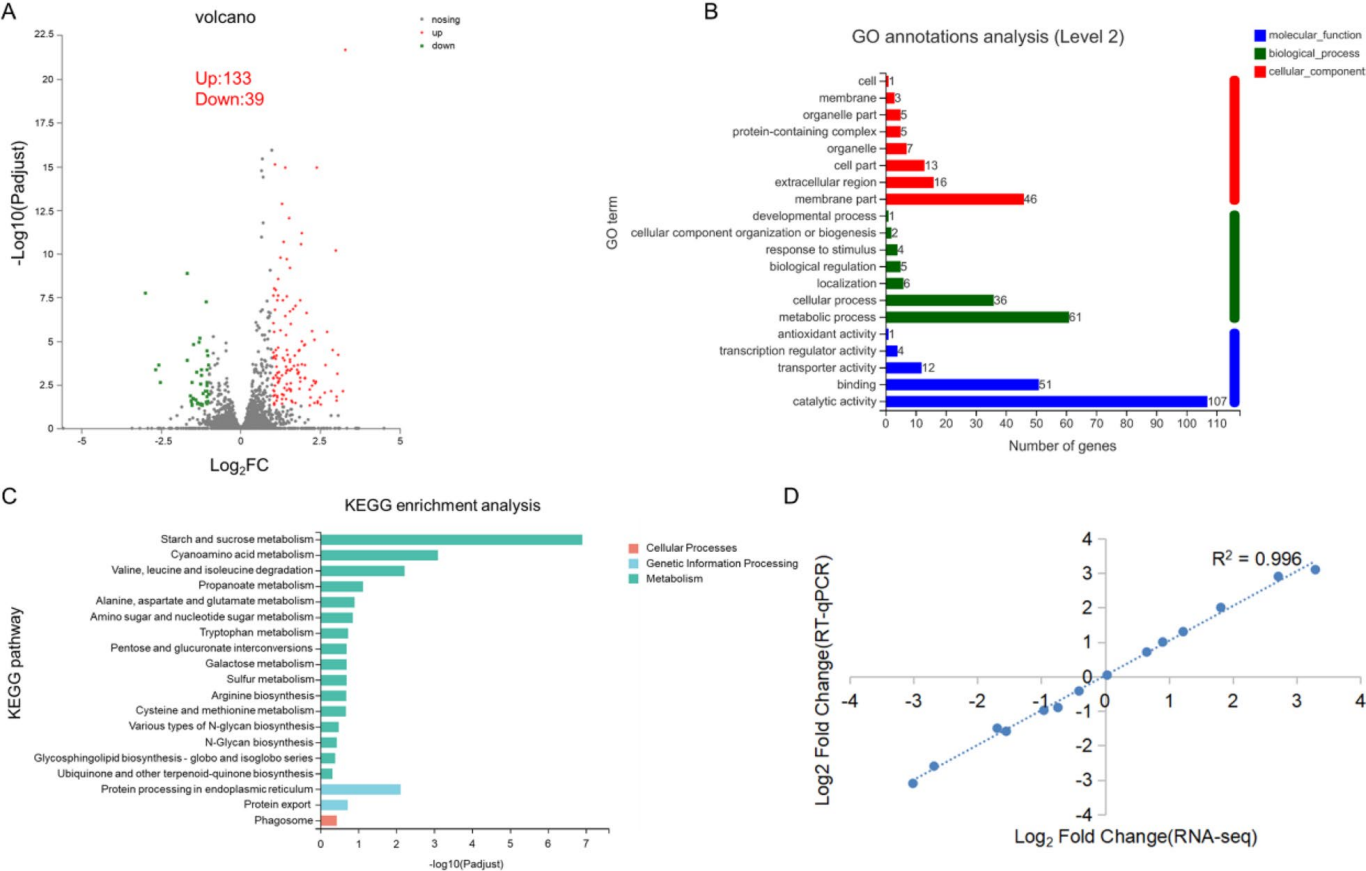
Transcriptome analysis results. **A** Volcano plot of the differential level of gene expression. The genes that were significantly upregulated and downregulated are denoted by red and green dots, respectively. Gray dots indicate genes whose expression was not significantly changed. **B** Gene Ontology (GO) annotations analysis of differentially expressed genes in the Δ*rsr1* and QM6a strains. Significantly enriched categories are shown (adjusted *P* < 0.05). **C** KEGG enrichment analysis for the Δ*rsr1* and QM6a strains. **D** Correlation between RT-qPCR and RNA sequencing results

To further understand the role of *rsr1* in cellulase production, we compared the two transcriptomes of the deletion strain (Δ*rsr1*) and the original strain (QM6a) using 1% (w/v) Avicel as the carbon source. Volcano analysis revealed significantly different expression of 172 genes (Log_2_fold change ≥ 1 or ≤  − 1 and adjusted *P* < 0.05; Fig. 7A), of which 133 were upregulated and 39 were downregulated (Δ*rsr1*_vs_QM6a; Additional file 5: Table S2). Gene ontology (GO) annotation analysis revealed different expression genes on metabolic processes, binding, catalytic activity, membrane part, cellular process, and other life activities (Fig. 7B). Kyoto Encyclopedia of Genes and Genomes (KEGG) enrichment analysis revealed the participation of different expression genes in starch and sucrose metabolism, cyanoamino acid metabolism, the degradation of valine, leucine, and isoleucine, and protein processing in endoplasmic reticulum pathways (Fig. 7C). This suggested that *rsr1* is a widely involved upstream signal that maintains the downstream switch ‘on’ because its deletion affects many pathways. This further suggested that *rsr1* influences the entire physiological process, which is very important for cellulase production.

Comparison of cellulase and hemicellulase gene expression levels in Δ*rsr1* and QM6a (Additional file 6: Table S3) revealed that cellulase and xylanase gene expression levels were upregulated. Of these, the expression levels of endoglucanases (EGL1, EGL3, and EGL5), swollenin, and xylanase *xyn4* were highly upregulated (approximately 4–eightfold), consistent with our RT-qPCR data (Fig. 7D). These data indicated that *rsr1* was involved in the negative regulation of cellulase expression during induction. We then searched for the top 10 significantly upregulated and downregulated genes (Additional file 7: Table S4). The top 10 upregulated genes included endoglucanase (TRIREDRAFT_122081; *egl1*) and exo-1,4-beta-xylosidase bxlB (TRIREDRAFT_58450). The expression levels of the 12 transcription factors involved in cellulase expression are listed in Additional file 8: Table S5. The transcript abundance of the cellulase activator genes *ace3* and *xyr1* were significantly upregulated by 1.73- and 0.7-fold in Δ*rsr1*, respectively (Table 2), compared to the parental strain QM6a. This indicates their positive effect on the improvement of cellulase production in Δ*rsr1*. These results suggested that deletion of *rsr1* greatly increases expression of cellulase-related genes.

**Table 2:** Log_2_ fold change (Log_2_fc) of characterized transcriptional factors involved in the regulation of lignocellulase genes in Δ*rsr1* and QM6a strains

Gene ID	Transcription factor genes	Log_2_fc	Up/Down	Positive/Negative-acting	Adjusted *P*
77513	ACE3	1.45	up	Positive	2.07E-10
122208	XYR1	0.77	up	Positive	6.40E-03
52368	BglR	0.37	up	Positive	3.94E-03
76817	AreA	0.35	up	Positive	4.98E-02

Previous studies have shown that sugar transporters play an important role in the induction of cellulase [46, 47]. A total of 876 predicted transporter genes in *T. reesei* QM6a genome (https://mycocosm.jgi.doe.gov/mycocosm/annotations/browser/tcdb/summary;AtD9I7?p=Trire2) were selected (Additional file 9: Table S6), of which 22 were differentially expressed (adjusted *P* < 0.05) (Table 3) in Δ*rsr1* compared to QM6a. The MFS transporter (TRIREDRAFT_66228) and MFS general substrate transporter (TRIREDRAFT_70933) were significantly upregulated by 7.06- and 3.53-fold, respectively.

**Table 3:** Log_2_ fold change (Log_2_fc) of characterized transporters in Δ*rsr1* and QM6a strains

Gene ID	Description	Log_2_fc	Up/Down	Adjusted *P*
66999	AMP-binding enzyme	3.07	Up	6.06E-05
66228	MFS transporter	3.01	Up	2.54E-02
106936	Ferric reductase	2.99	Up	6.77E-11
55049	Casein kinase 1, delta subunit	2.50	Up	1.71E-02
70933	MFS general substrate transporter	2.18	Up	4.99E-02
48444	MFS maltose permease	1.90	Up	2.86E-11
56684	Sugar (and other) transporter	1.81	Up	3.81E-05
46819	Sugar (and other) transporter	1.55	Up	1.50E-04
104072	Hexose transporter	1.46	Up	3.42E-05
50894	Sugar (and other) transporter	1.45	Up	2.90E-03
122920	Hsp70 protein	1.44	Up	2.70E-08
43974	UAA transporter	1.32	Up	4.61E-08
60945	Sugar (and other) transporter	1.30	Up	3.99E-02
62171	MFS permease	1.22	Up	1.42E-03
122048	Sec61beta family	1.03	Up	2.53E-08
69957	Maltose permease	1.02	Up	4.32E-05
55240	Large-conductance mechanosensitive channel, MscL	− 1.05	Down	6.75E-05
59364	OPT-domain-containing protein	− 1.24	Down	9.31E-04
5502	Aldo/keto reductase	− 1.27	Down	4.40E-02
54694	Oxidoreductase	− 1.36	Down	3.99E-02
57015	Transmembrane amino acid transporter	− 1.53	Down	4.66E-02
76880	RAS-GTPase RSR1	− 3.01	Down	1.78E-08

### Connection between RSR1 and GPCRs

GPCRs receive extracellular signals that may be transmitted through the Ras small GTPases [22]. Only three of 58 GPCR genes (Additional file 10: Table S7) showed significantly increased transcriptional levels in the RSR1 deletion strain compared to the wild-type strain (Table 4). The expression of VII GPCR (TRIREDRAFT_53238) was upregulated by 1.23-fold, while the expression of the two PTH11-like GPCRs (TRIREDRAFT_58767 and TRIREDRAFT_62462) was upregulated by 1.70- and 1.40- fold, respectively.

**Table 4:** Log_2_ fold change (Log_2_fc) of G-protein coupled receptors in Δ*rsr1* and QM6a strains

Gene ID	GPCR class	Log_2_fc	Adjusted* P*	Up/Down
53238	VII (related to rat growth hormone releasing factor)	1.16	7.06E-04	Up
58767	PTH11-like GPCRs	1.43	7.07E-04	Up
62462	PTH11-like GPCRs	1.26	1.60E-10	Up

*GPCR* G-protein coupled receptor

**Fig. 8:**
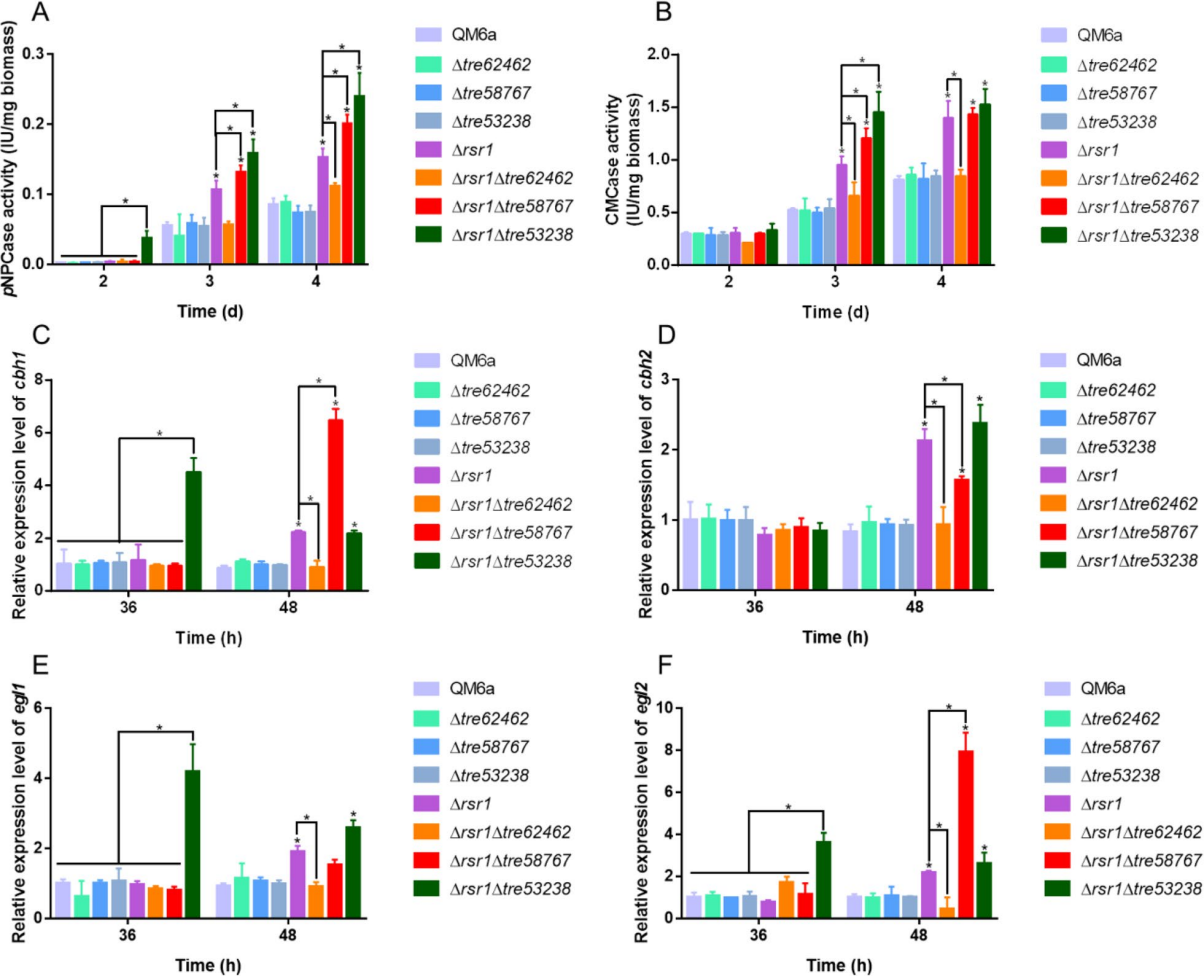
The role of three G-protein coupled receptors (GPCRs) on *rsr1*-mediated cellulase overproduction. *p*NPCase activity (**A**) and CMCase activity (**B**) of *T. reesei* QM6a, Δ*tre62462*, Δ*tre58767*, Δ*tre53238*, Δ*rsr1*, Δ*rsr1*Δ*tre62462*, Δ*rsr1*Δ*tre58767*, and Δ*rsr1*Δ*tre53238* cultured in MA with 1% (w/v) Avicel at 2, 3, and 4 d. The transcript levels of *cbh1* (**C**), *cbh2* (**D**), *egl1* (**E**) and *egl2* (**F**) in *T. reesei* QM6a, Δ*tre62462*, Δ*tre58767*, Δ*tre53238*, Δ*rsr1*, Δ*rsr1*Δ*tre62462*, Δ*rsr1*Δ*tre58767*, and Δ*rsr1*Δ*tre53238* strains cultured in MA with 1% (w/v) Avicel at 36 and 48 h. Values are represented as mean ± SD of the results from three independent experiments. Asterisks (*) indicate significant differences compared to the parental strain (Student’s *t*-test, *P < 0.05)

To further investigate the roles of three transcriptionally elevated GPCRs in *rsr1* deletion-mediated cellulase overexpression, double knockout strains (Δ*rsr1*Δ*tre62462*, Δ*rsr1*Δ*tre58767*, and Δ*rsr1*Δ*tre53238*) were constructed. Figure 8A, B show that loss of *tre58767* and *tre53238* can lead to an even higher level of cellulase production than that of the parental strain, while the loss of *tre62462* had the opposite effect. We then examined the transcript levels of cellulase-encoding genes of all strains by RT-qPCR (Fig. 8C–F), consistent with previous enzyme activity data (Fig. 8A, B). The Δ*rsr1*Δ*tre58767* strain exhibited promotion of the transcript of *cbh1* and *egl2*, while the Δ*rsr1*Δ*tre53238* strain appeared to promote the expression of *cbh1*, *egl1*, and *egl2*. The expression levels of the main cellulase genes in Δ*rsr1*Δ*tre62462* decreased by > 50% compared to Δ*rsr1* at 48 h. Moreover, we found that cellulase synthesis and gene regulation was not affected by the deletion of *tre62462*, *tre58767*, and *tre53238* (Fig. 8A–F). The results suggest that RSR1 and GPCRs may be connected and that they are involved in a complex signal transmission pathway.

## Discussion

The Ras subfamily of the small GTPase family is one of the most highly researched protein families [48]. In this study, 11 putative Ras subfamily genes were knocked out to investigate their involvement in cellulase signal transduction (Table 1). Three genes (*tre107035*, *tre66480*, and *tre70548*) could not be knocked out, indicating that they might be indispensable for growth. In our study, the deletion of *TrRas1* had no effect on cellulase production, while the loss of *TrRas2* showed a negative effect on cellulase production. These findings are consistent with those of a previous study by Zhang et al. [37]. In the present study, the loss of *rsr1* led to a remarkable increase in *p*NPCase, CMCase, FPase, and xylanase activities, and extracellular protein production (Fig. 1A–F). RT-qPCR analysis showed that the transcription levels of four major cellulases and two major xylanase genes (*cbh1*, *cbh2*, *egl1*, *egl2*, *xyn1*, and *xyn2*) were greatly improved (Fig. 2A–F), which is consistent with the enzyme activity data (Fig. 1A–F). The results illustrated that RSR1 has a negative effect on cellulase and xylanase production. Phylogenetic analysis revealed the widespread presence of *rsr1* in Ascomycota (Fig. 4B). These findings indicated that RSR1 homologs perform conserved functions in these filamentous fungi. Rsr1 GTPase interacts with Cdc42 and its related proteins to direct the establishment of cell polarity at the proper bud site in *S. cerevisiae* [49]. In *Aspergillus fumigatus*, the loss of *rsrA* (a *rsr1* homolog in *S. cerevisiae*) leads to a modest growth rate reduction and delay in germ tube emergence [50]. In *Ashbya gossypii*, *rsr1* is involved in hyphal growth guidance [51]. Moreover, Rsr1 was reported to participate in development regulation, aflatoxin biosynthesis and pathogenicity in *Aspergillus flavus* [52]. RSR1 has been widely reported in other fungi, but unfortunately its function in *T. reesei* remains elusive.

The deletion of *rsr1* significantly decreased the intracellular cAMP concentration (Fig. 5A) and transcription levels of ACY1-cAMP-PKA pathway genes (*acy1*, *pkac1*, *pkac2*, and *pkar1*) (Fig. 5C–F). Further, the deletion of *acy1* was favorable for cellulase production, while the overexpression of *acy1* was disadvantageous for cellulase production in Δ*rsr1*, indicating that the ACY1-cAMP-PKA pathway is involved in the *rsr1*-related signal transduction pathway (Fig. 6A–F). In a previous study, Chen et al. [53] demonstrated that deletion of *acy1* in *T. reesei* QM6a resulted in a significant decrease of enzymatic activity and the transcript abundance of the corresponding genes (*cbh1* and *egl1*) during growth on Avicel. In their study, conidia were fed with 2% glucose for 36–48 h to form mycelia and then transferred to 1% (w/v) Avicel [53]. When *acy1* was deleted, the *T. reesei* strain failed to respond to a change of the input signal from repression to induction, resulting in less cellulase production, which also shows that *acy1* may be a key signaling molecule in signal transmission. Schuster et al. [33] showed that *cbh1*/*cbh2* transcript levels in the parental strain QM9414 are similar to those of the *acy1-*deleted strain in the early stage in darkness, whereas we found that the *cbh1*/*cbh2* transcript levels in our ∆*acy1* strain were enhanced. This divergence may be related to the different parental strains (QM6a in our study) and different carbon sources. Nonetheless, our finds agree with those of Schuster et al. [33] to what concerns the increased *cbh1*/*cbh2* levels upon *pkac1* loss in the dark. Nogueira et al. [54] showed that addition of 1 mM dibutyryl-cAMP in *acy1*-deleted strain leads to increased cellulase CBH1 secretion, whereas in the parental strain QM9414 has the opposite result. This finding illustrates that cAMP regulates the secretion of cellulolytic enzymes in a dual manner. In *S. cerevisiae* and *C. albicans*, the Ras/cAMP/PKA pathway plays an important role in regulating physiological metabolism [31, 32]. *In F. graminearum*, the RasGEF FgCdc25 modulates the cAMP signaling pathway to regulate fungal development and invasiveness [25]. These studies suggest that the Ras/cAMP/PKA pathway is critical for fungi, including *T. reesei*.

The expression of cellulase genes needs to be stimulated by specific inducing carbon sources, such as Avicel or lactose. When these are recognized by the GPCRs or transporters on the surface of cells, the signals may be transmitted to the transcription factors by small GTPases through the ACY1-cAMP-PKA pathway [22, 23, 31, 32, 37, 55]. Here, transcriptomic analysis suggested that *rsr1* might connect to some GPCRs and transporters and negatively regulate the expression of crucial cellulase transcription activators, such as ACE3 and XYR1 (Table 2). Three GPCRs (TRIREDRAFT_53238, TRIREDRAFT_58767, and TRIREDRAFT_62462) might be involved in RSR1 signaling because they showed significantly increased transcriptional levels in the *rsr1* deletion strain (Table 4). A previous study reported that the TRIREDRAFT_53238 regulates secondary metabolism in *T. reesei* [56], indicating its regulatory role. Then, we used Δ*rsr1* as the starting strain to delete *tre62462*, *tre58767*, and *tre53238*. With the loss of *tre62462*, the cellulase enhancement mediated by the deletion of *rsr1* was effectively attenuated (Fig. 8A–F). Based on Δ*rsr1*, loss of *tre58767* and *tre53238* further promoted cellulase production (Fig. 8A–F). The results indicated that the three GPCRs may be involved in the *rsr1*-related signal transduction pathway in *T. reesei*. GPCR signaling can lead to the activation of Ras and Rho GTPases through heterotrimeric G-proteins [23, 55]; whether the extracellular signals received by GPCRs are transmitted directly or indirectly to Ras small GTPases in fungi has not been reported in detail. Therefore, further research is needed. A previous study showed that Ras-like small GTPases can also regulate GPCR anterograde trafficking, specifically the Rab and Sar1/ARF subfamilies which are well described as traffic cops to control cargo transport between various intracellular organelles [57]. Whether *rsr1* controls trafficking of GPCRs in *T. reesei* similar to other Ras GTPases remains unknown and should be investigated. Since the activity of Ras small GTPases is controlled by GEFs and GAPs [9], whether *rsr1* interacts with these regulatory proteins should be explored.

**Fig. 9:**
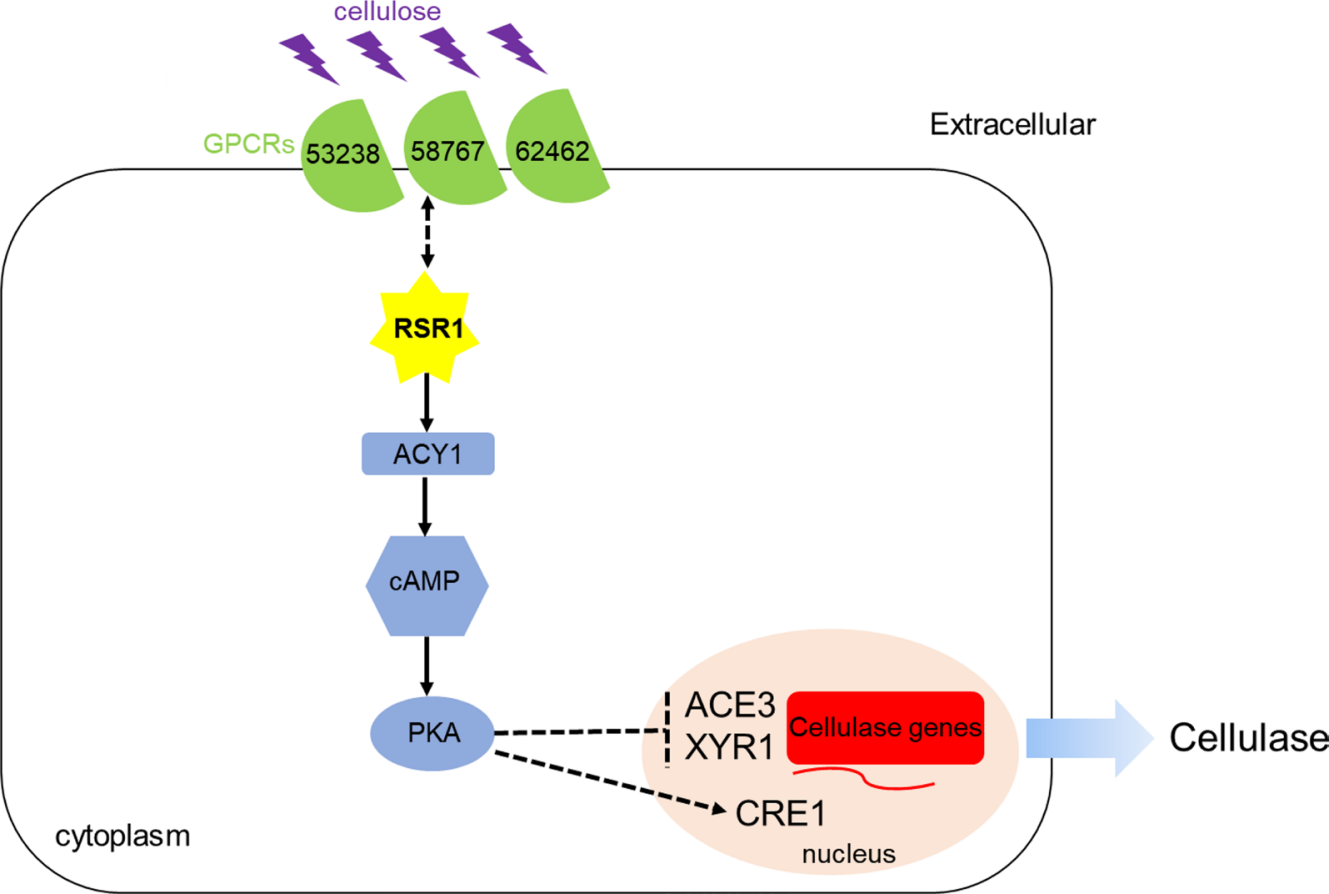
Putative mechanism for RSR1 regulating cellulase production in *T. reesei*. GPCRs are shaded in green, ACY1-cAMP-PKA pathway in blue, cellulose molecule in purple, cellulase gene in red, and RSR1 in yellow. Positive regulation is represented by a solid line with arrowheads. Negative regulation is represented by a line ending in a vertical line. Dotted arrows in both directions represent unknown connections. The solid arrows indicate data supported by our results, and the dashed arrows indicate undefined regulation. After receiving signal stimulation from extracellular cellulose molecules, three GPCRs on the membrane may transmit signals to RSR1, promoting the expression of ACY1-cAMP-PKA pathway-related genes, increasing intracellular cAMP content, negatively regulating the expression of ACE3 and XYR1, promoting the expression of CRE1, and ultimately repressing cellulase production

Our study showed that the deletion of *rsr1* can induce the expression of the crucial cellulase activators XYR1 and ACE3. We also found that the transcription level of the CCR repression transcription factor CRE1 decreased significantly (Additional file 2: Figure S1). The results implied that RSR1 may transmits signals from transporters (Table 3) or GPCRs (Table 4) to enhance *acy1* expression, intracellular cAMP synthesis, and activation of PKA kinases, then to negatively control activators, such as XYR1 and ACE3, and to induce repressors, such as CRE1 (Fig. 9). In *T. reesei*, *pkac1* is involved in negative modulation of the expression of cellulase genes by adjusting the expression of XYR1 [33]. In *Aspergillus nidulans*, the lack of *pkaA* genes affects the localization of CRE1 in the nucleus, leading to increased secretion of hydrolase [12]. How *pkac1* phosphorylates transcription factors, such as ACE3, XYR1, and CRE1 in *T. reesei* remains unknown, which is also worthy of further study.

Our findings demonstrated that the Ras small GTPase RSR1 negatively regulates cellulase expression through the ACY1-cAMP-PKA pathway. Previous studies have shown that *Ras2* promotes the expression of cellulase genes [37], while *rac1* acts as a negative regulator [58], suggesting that the pathway of action of small GTPases is a multichannel circuit. In the future, overexpression of the *Ras2* gene or knockout of *rac1* in the RSR1 deletion strain will be conducted to determine whether there are interactions between the three small GTPases.

## Conclusions

In summary, the deletion of Ras small GTPase RSR1 significantly enhanced cellulase production in the wild-type strain and cellulase hyper-producer RUT-C30. With the deletion of RSR1, we found the upregulation of the ACE3 and XYR1 transcriptional activators. Loss of RSR1 also significantly reduced the expression of ACY1-cAMP-PKA pathway genes and the content of intracellular cAMP. We conclude that RSR1 negatively regulates cellulase synthesis through the ACY1-cAMP-PKA pathway and that three upstream GPCRs may be involved in the *rsr1*-related signal transduction pathway. Our study revealed a putative mechanism for the regulation of cellulase production by Ras GTPase *rsr1* in *T. reesei* (Fig. 9). Further, our results may be useful for the construction of cellulase hyper-producers to solve the energy crisis. This study also provides new perspectives for understanding the mechanism of Ras small GTPase in regulating cellulase production in fungi.

## Methods

### Strains and culture conditions

*Escherichia coli* strain DH5α was used for plasmid construction. *T. reesei* QM6a (ATCC 13,631) and RUT-C30 (ATCC 56,765) were used as parental strains, while *Agrobacterium tumefaciens* GV3101 was used to transform the *T. reesei* strains [59]. The fungal strains were grown on potato dextrose agar with 2% (w/v) glucose as the carbon source at 28 °C for 6 d, and then the conidia were washed with 20% glycerol and stored at − 40 ℃. All fungal strains were cultivated in the dark.

To analyze enzyme production, the conidia (final concentration 10^6^ mL^−1^) of *T. reesei* strains were placed in a 250-mL bottle with 40 mL MA liquid medium [18] containing 1% (w/v) Avicel (PH-101, Sigma-Aldrich, St. Louis, MO, USA), 2% (w/v) lactose, or 2% (w/v) glucose as the sole carbon source, and incubated at 28 °C in a dark incubator with a rotation speed of 220 rpm. Hyphae (3 mL) were collected at 36, 48, and 60 h, pressed dry with filter paper, quick-frozen in liquid nitrogen, and kept frozen at − 80 °C for RNA extraction. The culture medium (1 mL) was removed, centrifuged at 12,000 × *g* for 10 min, and the supernatant was used for enzyme activity determination.

### Construction of plasmids and strains

To construct a *rsr1* deletion mutant, the 758-bp upstream and 701-bp downstream fragments of the gene encoding RSR1 were generated from the genome of *T. reesei* QM6a using KOD-Plus-Neo (TOYOBO, Osaka, Japan) with the primer pairs *rsr1*-D1/*rsr1*-D2 and *rsr1*-D3/*rsr1*-D4, respectively. As shown in Additional file 4: Fig S3, the upstream fragment was ligated into the *Pac*I and *Xba*I linearized LML2.1 [60] using the ClonExpress™ II One Step Cloning Kit (Vazyme, Nanjing, China) to form pF*rsr1*. Subsequently, the downstream fragment was inserted into *Swa*I-linearized pF*rsr1* to form the recombinant knockout plasmid pΔ*rsr1*. The knockout plasmids for the other 10 genes were constructed similarly; the knockout verification primers are listed in Additional file 11: Table S8. The deletion cassettes were transformed into *T. reesei* by *Agrobacterium*-mediated transformation [59]. The *rsr1* deletion strain (Δ*rsr1*) generated by double crossover was verified by diagnostic PCR using the primer pairs *rsr1*-CF/D70-4, Hg3.6/*rsr1*-CR, and *rsr1*-OF/ *rsr1*-OR with DNA sequencing (Additional file 4: Fig S3). Xylose-induced Cre recombinase was used to excise the hygromycin resistance cassettes prior to further analysis, as previously described [60]. To complement the *rsr1* in knockout strains, 3245-bp DNA fragments, including the 1.5-kb promoter, gene coding sequence, and 0.5-kb terminator, were amplified from the *T. reesei* genome using rc-*rsr1*-1/rc-*rsr1*-2 and rc-*rsr1*-3/rc-*rsr1*-4, and inserted into *Swa*I-linearized LML2.1 to generate the re-complementation vector pR*rsr1* (Additional file 4: Fig S3). The complementation cassette was transformed into the Δ*rsr1* strain by *Agrobacterium*-mediated transformation. The complementation strain (RC-*rsr1*) was screened using PCR and DNA sequencing (Additional file 4: Fig S3). The deletion vectors (pΔ*acy1*, pOE*acy1*, pΔ*tre62462*, p*tre58767*, and pΔ*tre53238*) were constructed following the principles above and subsequently transformed into the *T. reesei* QM6a and Δ*rsr1* strains using *Agrobacterium*-mediated transformation, respectively (Additional file 3: Fig S2). The verification method of transformants was the same as described above. The strains and primers used in this study are listed in Additional file 1: Table S1 and Additional file 11: Table S8, respectively.

### Phylogenetic analysis

The protein sequence of RSR1 was downloaded from the Joint Genome Institute of the Department of Energy (USDOE-JGI) website (http://genome.jgipsf.org/Trire2/). BLASTP (http://blast.ncbi.nlm.nih.gov/) was used to identify homologous proteins of RSR1 with an E value < 10^−5^ applied as a cutoff. MEGA7 software was used to perform phylogenetic analysis of proteins using a maximum likelihood tree with 1,000 bootstrap replicates [61].

### Enzymatic activity assays

The cellobiohydrolase activity of *p*NPCase was determined using 5 mM *p*-nitrophenol-D-cellobioside (Sigma-Aldrich) as a substrate in 50 mM sodium acetate buffer (pH 5.0) at 50 °C for 30 min [62]. The release of *p*-nitrophenol was assessed by measuring the absorbance at 405 nm. One unit of enzymatic activity was defined as 1 μmol of *p*-nitrophenol released from the substrate per min. CMCase, representing endoglucanase activity, was measured using 1% carboxymethylcellulose (CMC, Sigma-Aldrich) in 50 mM sodium acetate buffer (pH 5.0) at 50 °C for 30 min at an absorbance of 540 nm [63]. One unit of CMCase activity was defined as the amount of enzyme producing 1 μmol of reducing sugar per minute. The total cellulase activity was characterized by FPase activity, which was measured using Whatman filter paper as a substrate in 0.2 mL of 50 mM sodium acetate buffer (pH 5.0) at 50 °C for 60 min at an absorbance of 540 nm [64]. One unit of FPase activity was the amount of cellulase required to release 1 μmol of glucose within 1 min. Xylanase activity was measured using approximately 0.003 g xylan as a substrate in 0.2 mL of 50 mM sodium acetate buffer (pH 5.0) at 50 °C for 60 min at an absorbance of 540 nm [65]. One unit of xylanase activity was defined as the production of 1 μmol of reducing sugar per min. Protein concentrations were determined using a BCA Protein Assay Kit (Beyotime, Shanghai, China). Biomass was measured indirectly by measuring intracellular protein concentration at an absorbance of 562 nm, and biomass weight was calculated as 0.32 g intracellular protein per gram cell dry weight [66]. SDS-PAGE analysis of extracellular proteins was conducted on 12% Tris–HCl polyacrylamide gels using 20 μL of cell cultures after 3 d of cultivation.

### RNA isolation and RT-qPCR

The levels of gene-specific mRNA were assessed using RT-qPCR based on our previous research with some modifications [67]. Briefly, the total RNA from pre-frozen mycelia was extracted carefully using a FastRNA Pro Red Kit (MPbio, Irvine, CA, USA), according to the manufacturer’s instructions. The extracted RNA (500 ng) was reverse transcribed to cDNA using the PrimeScript RT Reagent Kit with gDNA eraser (TaKaRa Bio, Shiga, Japan), following the manufacturer’s instructions. Quantitative PCR was conducted using the TransStart TipTop Green qPCR SuperMix (TransGen, Shanghai, China) with 200 nM of forward and reverse primers (Additional file 11: Table S8). SYBR green assays along with ABI StepOne thermocycler (Applied Biosystems, Foster City, CA, USA) were used to analyze gene transcriptional levels using the 2^−ΔΔCt^ method. Gene expression levels were normalized with the reference genes *sar1* [64, 68, 69].

### Determination of intracellular cAMP content

The cultured hyphae were collected, quickly chilled with liquid nitrogen, and frozen at − 80 °C. The extraction and determination of intracellular cAMP was conducted as described previously [53, 70], with some modifications. Briefly, the collected hyphae were ground into a powder with liquid nitrogen and dissolved in phosphate-buffered saline (PBS, pH 7.4). After centrifugation, the supernatant was diluted and cAMP content was evaluated with the microorganism cAMP ELISA Kit (mlbio, Shanghai, China), according to the manufacturer’s protocol. The BCA Protein Assay Kit (Beyotime, Shanghai, China) was used to determine the total protein content of each sample. The intracellular cAMP content/intracellular protein concentrations were calculated.

### Transcriptome analysis

The same amount of conidia (final concentration 10^6^ mL^−1^) was cultivated at 28 °C (220 rpm) in 40 mL MA liquid medium supplemented with 1% (w/v) Avicel as the sole carbon source for 48 h. Three replicate samples of cultured hyphae were subsequently collected. The three parallel samples of QM6a and Δ*rsr1* were sent to Shanghai Majorbio Bio-pharm Technology Co., Ltd. (Shanghai, China) for preparation and RNA sequencing using the Illumina Novaseq 6000 sequencing platform (Illumina, San Diego, CA, USA). The original data after quality control, namely clean data (reads), were compared with the reference genome to obtain mapped data (reads) for subsequent transcript assembly and expression calculation. The transcriptome was sequenced simultaneously for comparison. Quality evaluation of the results mainly included sequencing saturation, gene coverage, distribution of reads in different regions of the reference genome, and analysis of the distribution of reads among different chromosomes. The following link (http://fungi.ensembl.org/Trichoderma_reesei/Info/Index) was used as the reference genome during the RNA sequencing process [18]. The expression levels of transcripts were quantitatively analyzed by the software RSEM (http://deweylab.github.io/RSEM/) [71] with transcripts per million reads as the quantitative index. DESeq2 was used to analyze raw counts and for differential expression analysis. FDR correction with Benjamini/Hochberg was used in this study to control the probability or frequency of errors in the overall inference results and to correct the* P* value to adjusted *P*. Genes whose adjusted *P* was < 0.05 and whose |log_2_fold change|≥ 1 were selected as differentially expressed genes. Raw whole transcriptome shotgun sequencing data can be found on the NCBI SRA website (https://www.ncbi.nlm.nih.gov/sra/PRJNA888813).

Each independent sample was processed meticulously, with no significant difference between parallel samples (Additional file 12: Fig S4). The clean reads of each sample were compared with the designated reference genome. The comparison rate ranged from 96.49 to 97.03%. The correlation between the three biological replicates of the parent and deletion strains used in the transcriptional analysis was very high (Pearson correlation, r^2^ ≥ 0.8487; Additional file 12: Fig S4). To verify the reliability of transcriptome data, 14 genes were randomly selected, and their mRNA expression levels were detected by RT-qPCR. There was a significant Pearson correlation between the transcriptome and RT-qPCR data (R^2^ = 0.996), indicating that the transcriptome analysis was reliable (Fig. 7D).

### Statistical analysis

All experimental data were obtained from at least three independent samples with the same or similar results. Standard deviations from the mean of triplicate determinations are indicated by error bars. Student’s *t*-test was used to evaluate differences between the sample groups. Multiple comparisons were performed using Duncan’s multiple-range test. The results were significantly different if *P* was < 0.05.

## Supplementary Information

**Additional file 1: Table S1.** *T. reesei* strains constructed in this study

**Additional file 2: Figure S1.** The effect of rsr1 on CCR. pNPCase activity (A) of the three strains (QM6a, Δrsr1, and RC-rsr1) were measured, complementing with 2% (w/v) glucose as the sole carbon source. The cre1 transcriptional levels (B) of the three strains (QM6a, Δrsr1, and RC-rsr1) were measured, complementing with 1% (w/v) Avicel as the sole carbon source. Asterisks (*) indicate significant differences compared to parental strain (Student’s t-test, ***P < 0.001; ****P < 0.0001; ns means no significant difference)

**Additional file 3: Figure S2.** The effect of* rsr1* on hyphal growth of *T. reesei* strains. A. Three strains (QM6a, Δ*rsr1*, and RC-*rsr1*) were cultured on PDA medium with 2% glucose at 28 °C in the dark and then photos were taken at 3 days. B. RUT-C30 and C30-*rsr1 *were cultured on PDA medium with 2% glucose at 28 °C in the dark and then photos were taken at 3 days.

**Additional file 4: Figure S3.** Construction and verification of deletion mutants. A. To construct an *rsr1* deletion mutant, the 758-bp upstream and 701-bp downstream fragments of *rsr1* were amplified from *T. reesei* QM6a genomic DNA were amplified by PCR. Then, upstream and downstream fragments were attached to the *Pac*I/*Xba*I and *Swa*I sites in an orderly manner, respectively, of linearized LML2.1 [1], to form the* rsr1* deletion plasmid (p∆*rsr1*). The deletion cassettes were transformed into QM6a strain by *Agrobacterium*-mediated transformation. The primers *rsr1*-CF/D70-4, Hg3.6/*rsr1*-CR and *rsr1*-OF/ *rsr1*-OR were used to verify the putative *rsr1* disruption mutants (Δ*rsr1*) by diagnostic PCR using with DNA sequencing. Hyg casseette were used as screening markers. B. To construct an RSR1 complementation strain, the 1.5-kb promoter fragment, gene coding sequence fragment, and 0.5-kb terminator fragment together were obtained from the *T. reesei genome* QM6a, which constituted 3245-bp and inserted into *Swa*I-linearized LML2.1 to turn into the re-complementation vector pR*rsr1* [2]. The re-complementation cassettes were transformed into Δ*rsr1* strain by *Agrobacterium*-mediated transformation. Re-complementation strains (RC-*rsr1*) are screened by PCR using the primers rc-*rsr1*-CF and rc-*rsr1*-CR and DNA sequencing. The primers were used in this experiment in Table S3. C. Validated electrophoretic diagram to verify the knockout and complementation of *rsr1*. D. The skeleton schematic diagram to knockout *acy1* in the knockout strain Δ*rsr1*. E. The skeleton schematic diagram to overexpress *acy1* in the knockout strain Δ*rsr1*. F. The skeleton schematic diagram to knockout *tre62462* in the knockout strain Δ*rsr1*. G. The skeleton schematic diagram to knockout *tre58767* in the knockout strain Δ*rsr1*. H. The skeleton schematic diagram to knockout *tre53238* in the knockout strain Δ*rsr1*. I. Validated electrophoretic diagram to verify the deletion of* tre62462*, *tre58767*, *tre53238*, *acy1 *in the knockout strain Δ*rsr1*, and the overexpression of *acy1* in the knockout strain Δ*rsr1*. 1: ∆*rsr1*∆*tre62462*-F; 2: ∆*rsr1*∆*tre62462*-R; 3: ∆*rsr1*∆*tre58767*-F; 4: ∆*rsr1*∆*tre58767*-R; 5: ∆*rsr1*∆*tre53238*-F; 6: ∆*rsr1*∆*tre53238*-R; 7: ∆*rsr1*∆*acy1*-F; 8: ∆*rsr1*∆*acy1*-R; 9: ∆*rsr1*-OE*acy1*-F; 10: ∆*rsr1*-OE*acy1*-R. J. Validated electrophoretic diagram to verify the deletion of* acy1 *in the original strain QM6a, and the overexpression of *acy1 *in the original strain QM6a. 1: ∆*acy1*-F; 2: ∆*acy1*-R; 3: ∆*acy1*-O; 4: QM6a-O; 5: QM6a-OE*acy1*-F; 6: QM6a-OE*acy1*-R. K. Validated electrophoretic diagram to verify the deletion of* tre62462*,* tre58767*, and *tre53238 *in the original strain QM6a. 1: ∆*tre62462*-F; 2: ∆*tre62462*-R; 3: ∆*tre62462*-O; 4: QM6a-O; 5: ∆*tre58767*-F; 6: ∆*tre58767*-R; 7: ∆*tre58767*-O; 8: QM6a-O; 9: ∆*tre53238*-F; 10: ∆*tre53238*-R; 11: ∆*tre53238*-O; 12: QM6a-O.

**Additional file 5: Table S2.** Transcriptional level of total DEGs in Δ*rsr1* and QM6a strains.

**Additional file 6: Table S3. **Comparison of cellulase and hemicellulase genes expression levels in Δ*rsr1* and QM6a strains

**Additional file 7: Table S4. **Log_2_ fold change (Log_2_fc) of the top 10 genes differentially expressed in Δ*rsr1* and QM6a strains

**Additional file 8: Table S5. **Log_2_fold change (Log_2_fc) of characterized transcriptional factors involved in the regulation of lignocellulase genes. NS represented not significant, *P* adjust > 0.05

**Additional file 9.Table S6** The changes of 876 predicted transporter genes in T. reesei QM6a genome were sorted out

**Additional file 10: Table S7. **The changes of 58 GPCR genes [1] in Δ*rsr1 *and QM6a strains were sorted out, NS represented not significant, P adjust > 0.05

**Additional file 11: Table S8 **Primers used in this study

**Additional file 12: Figure S4. **The correlation between the biological replicates of each sample. The following heat map is used to show the correlation. Each value in the grid is the correlation coefficient between two samples. The larger the value, the greater the correlation between the two samples and the closer they are. These results indicate that the transcriptome data is more credible

## Data Availability

All data generated or analyzed during this study are included in this published article**.**

## Abbreviations

cAMP: Cyclic adenosine monophosphate
CCR: Carbon catabolite repression
CMCase: Representing *endo*-β-glucanase activity
FPase: Filter paper activity, representing total extracellular cellulase activity
GPCR: G-protein coupled receptor
GTP: Guanosine triphosphate
GDP: Guanosine diphosphate
GEF: Guanine nucleotide exchange factor
GAP: GTPase activation protein
ACY1: Adenylate cyclase 1
PKA: Protein kinase A
*p*NPCase: Representing exo-β-glucanase activity
SDS-PAGE: Sodium dodecyl sulfate polyacrylamide gel electrophoresis
RT-qPCR: Real-time quantitative polymerase chain reaction
GO: Gene ontology
