# Supplementary material for: The Ras small GTPase RSR1 regulates cellulase production in Trichoderma reesei
Source: Biotechnol Biofuels Bioprod. 2023 May 23;16:87. doi: 10.1186/s13068-023-02341-z (PMC10204303; doi:10.1186/s13068-023-02341-z)
Supplement: Supplementary file 1 — Additional file 1: Table S1. T. reesei strains constructed in this study [file 13068_2023_2341_MOESM1_ESM.docx]

**Table S1** *T. reesei* strains constructed in this study

| Strains | Description | Source |
| --- | --- | --- |
| *T. reesei* QM6a | Parental strain | ATCC |
| *T. reesei* RUT-C30 | Parental strain | ATCC |
| Δ*TrRas1* | QM6a-deleted *TrRas1* gene | This study |
| Δ*TrRas2* | QM6a-deleted *TrRas2* gene | This study |
| Δ*rsr1* | QM6a-deleted *rsr1* gene | This study |
| Δ*tre107035* | QM6a-deleted *tre107035* gene | This study |
| Δ*tre66480* | QM6a-deleted *tre66480* gene | This study |
| Δ*tre34726* | QM6a-deleted *tre34726* gene | This study |
| Δ*tre107369* | QM6a-deleted *tre107369* gene | This study |
| Δ*tre67275* | QM6a-deleted *tre67275* gene | This study |
| Δ*tre70548* | QM6a-deleted *tre70548* gene | This study |
| Δ*tre61408* | QM6a-deleted *tre61408* gene | This study |
| Δ*tre81785* | QM6a-deleted *tre81785* gene | This study |
| C30-*rsr1* | RUT-C30-deleted *rsr1* gene | This study |
| RC*-rsr1* | Δ*rsr1*-harboring vector pRC-*rsr1* | This study |
| Δ*acy1* | QM6a-deleted *acy1* gene | This study |
| Δ*rsr1*Δ*acy1* | Δ*rsr1*-deleted *acy1* gene | This study |
| QM6a-OE*acy1* | QM6a-harboring vector pOE*acy1* | This study |
| Δ*rsr1*-OE*acy1* | Δ*rsr1*-harboring vector pOE*acy1* | This study |
| Δ*tre62462* | QM6a-deleted *tre62462* gene | This study |
| Δ*tre58767* | QM6a-deleted *tre58767* gene | This study |
| Δ*tre53238* | QM6a-deleted *tre53238* gene | This study |
| Δ*rsr1*Δ*tre62462* | Δ*rsr1*-deleted *tre62462* gene | This study |
| Δ*rsr1*Δ*tre58767* | Δ*rsr1*-deleted *tre58767* gene | This study |
| Δ*rsr1*Δ*tre53238* | Δ*rsr1*-deleted *tre53238* gene | This study |
