# Supplementary material for: The Ras small GTPase RSR1 regulates cellulase production in Trichoderma reesei
Source: Biotechnol Biofuels Bioprod. 2023 May 23;16:87. doi: 10.1186/s13068-023-02341-z (PMC10204303; doi:10.1186/s13068-023-02341-z)
Supplement: Supplementary file 2 — Additional file 2: Figure S1. The effect of rsr1 on CCR. pNPCase activity (A) of the three strains (QM6a, Δrsr1, and RC-rsr1) were measured, complementing with 2% (w/v) glucose as the sole carbon source. The cre1 transcriptional levels (B) of the three strains (QM6a, Δrsr1, and RC-rsr1) were measured, complementing with 1% (w/v) Avicel as the sole carbon source. Asterisks (*) indicate significant differences compared to parental strain (Student’s t-test, ***P < 0.001; ****P < 0.0001; ns means no significant difference) [file 13068_2023_2341_MOESM2_ESM.docx]

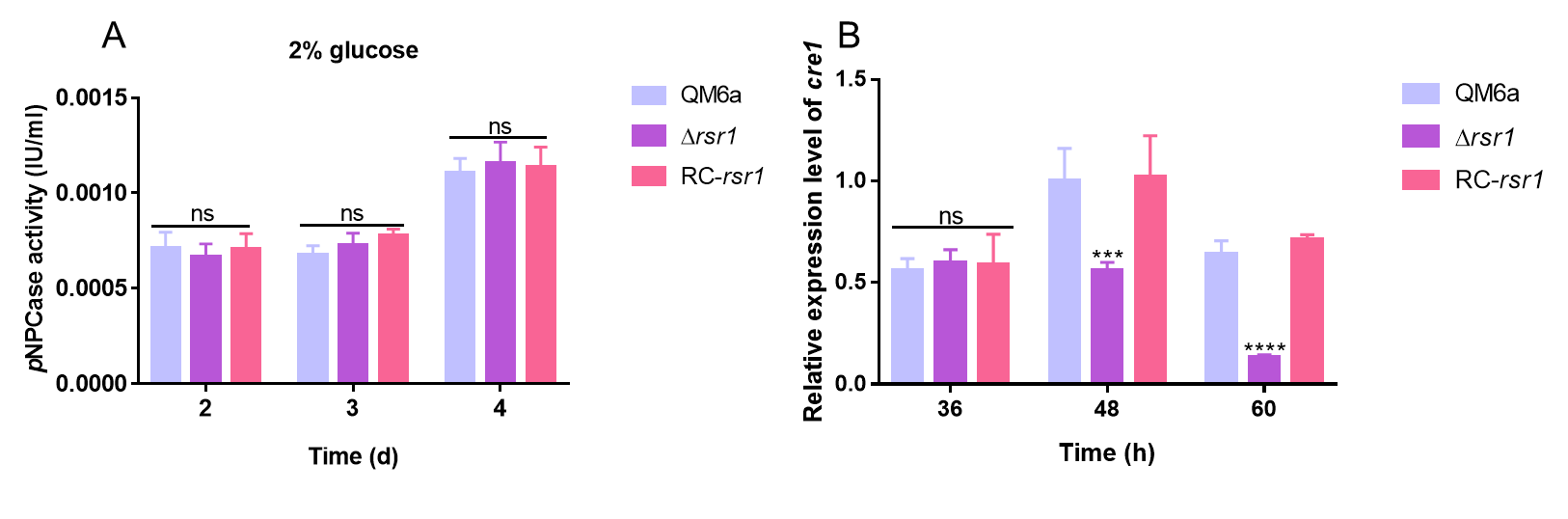


**Figure S1.** The effect of *rsr1* on CCR. *p*NPCase activity (A) of the three strains (QM6a, Δ*rsr1*, and RC-*rsr1*) were measured, complementing with 2% (w/v) glucose as the sole carbon source. The *cre1* transcriptional levels (B) of the three strains (QM6a, Δ*rsr1*, and RC-*rsr1*) were measured, complementing with 1% (w/v) Avicel as the sole carbon source. Asterisks (*) indicate significant differences compared to parental strain (Student’s *t*-test, ****P* < 0.001; *****P* < 0.0001; ns means no significant difference).
