# Supplementary material for: The Ras small GTPase RSR1 regulates cellulase production in Trichoderma reesei
Source: Biotechnol Biofuels Bioprod. 2023 May 23;16:87. doi: 10.1186/s13068-023-02341-z (PMC10204303; doi:10.1186/s13068-023-02341-z)
Supplement: Supplementary file 3 — Additional file 3: Figure S2. The effect of rsr1 on hyphal growth of T. reesei strains. A. Three strains (QM6a, Δrsr1, and RC-rsr1) were cultured on PDA medium with 2% glucose at 28 °C in the dark and then photos were taken at 3 days. B. RUT-C30 and C30-rsr1 were cultured on PDA medium with 2% glucose at 28 °C in the dark and then photos were taken at 3 days. [file 13068_2023_2341_MOESM3_ESM.docx]

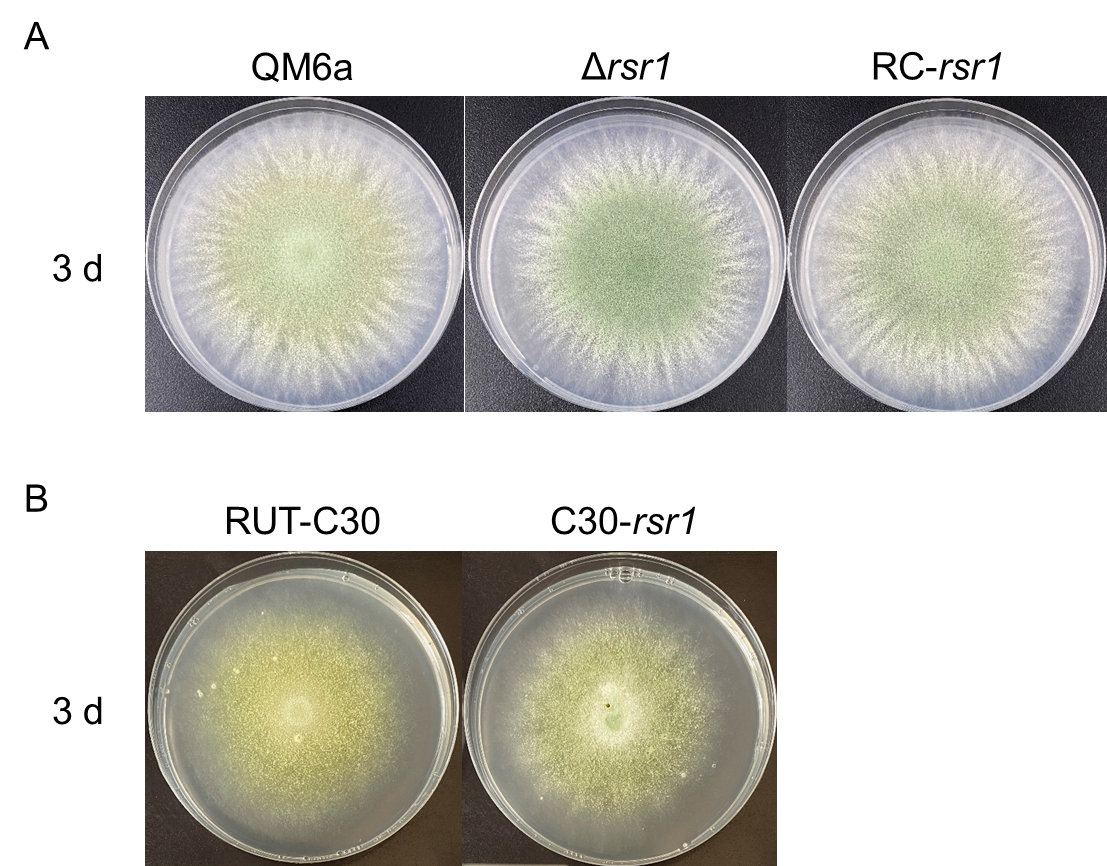


**Figure S2.** The effect of *rsr1* on hyphal growth of *T. reesei* strains. A. Three strains (QM6a, Δ*rsr1*, and RC-*rsr1*) were cultured on PDA medium with 2% glucose at 28 °C in the dark and then photos were taken at 3 d. B. RUT-C30 and C30-*rsr1* were cultured on PDA medium with 2% glucose at 28 °C in the dark and then photos were taken at 3 d.
