# Supplementary material for: The Ras small GTPase RSR1 regulates cellulase production in Trichoderma reesei
Source: Biotechnol Biofuels Bioprod. 2023 May 23;16:87. doi: 10.1186/s13068-023-02341-z (PMC10204303; doi:10.1186/s13068-023-02341-z)
Supplement: Supplementary file 4 — Additional file 4: Figure S3. Construction and verification of deletion mutants. A. To construct an rsr1 deletion mutant, the 758-bp upstream and 701-bp downstream fragments of rsr1 were amplified from T. reesei QM6a genomic DNA were amplified by PCR. Then, upstream and downstream fragments were attached to the PacI/XbaI and SwaI sites in an orderly manner, respectively, of linearized LML2.1 [1], to form the rsr1 deletion plasmid (p∆rsr1). The deletion cassettes were transformed into QM6a strain by Agrobacterium-mediated transformation. The primers rsr1-CF/D70-4, Hg3.6/rsr1-CR and rsr1-OF/ rsr1-OR were used to verify the putative rsr1 disruption mutants (Δrsr1) by diagnostic PCR using with DNA sequencing. Hyg casseette were used as screening markers. B. To construct an RSR1 complementation strain, the 1.5-kb promoter fragment, gene coding sequence fragment, and 0.5-kb terminator fragment together were obtained from the T. reesei genome QM6a, which constituted 3245-bp and inserted into SwaI-linearized LML2.1 to turn into the re-complementation vector pRrsr1 [2]. The re-complementation cassettes were transformed into Δrsr1 strain by Agrobacterium-mediated transformation. Re-complementation strains (RC-rsr1) are screened by PCR using the primers rc-rsr1-CF and rc-rsr1-CR and DNA sequencing. The primers were used in this experiment in Table S3. C. Validated electrophoretic diagram to verify the knockout and complementation of rsr1. D. The skeleton schematic diagram to knockout acy1 in the knockout strain Δrsr1. E. The skeleton schematic diagram to overexpress acy1 in the knockout strain Δrsr1. F. The skeleton schematic diagram to knockout tre62462 in the knockout strain Δrsr1. G. The skeleton schematic diagram to knockout tre58767 in the knockout strain Δrsr1. H. The skeleton schematic diagram to knockout tre53238 in the knockout strain Δrsr1. I. Validated electrophoretic diagram to verify the deletion of tre62462, tre58767, tre53238, acy1 in the knockout strain Δrsr1, [file 13068_2023_2341_MOESM4_ESM.docx]

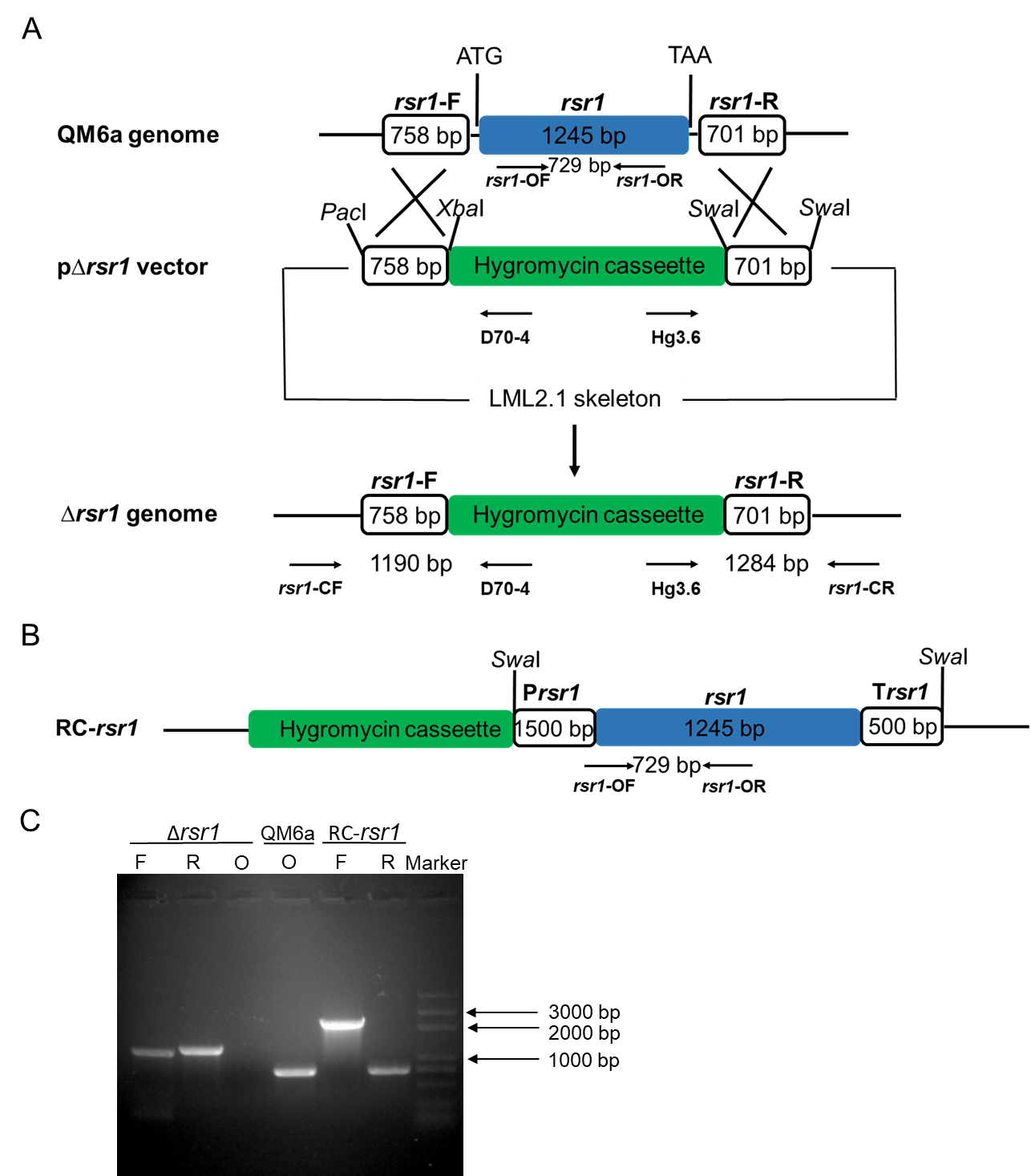


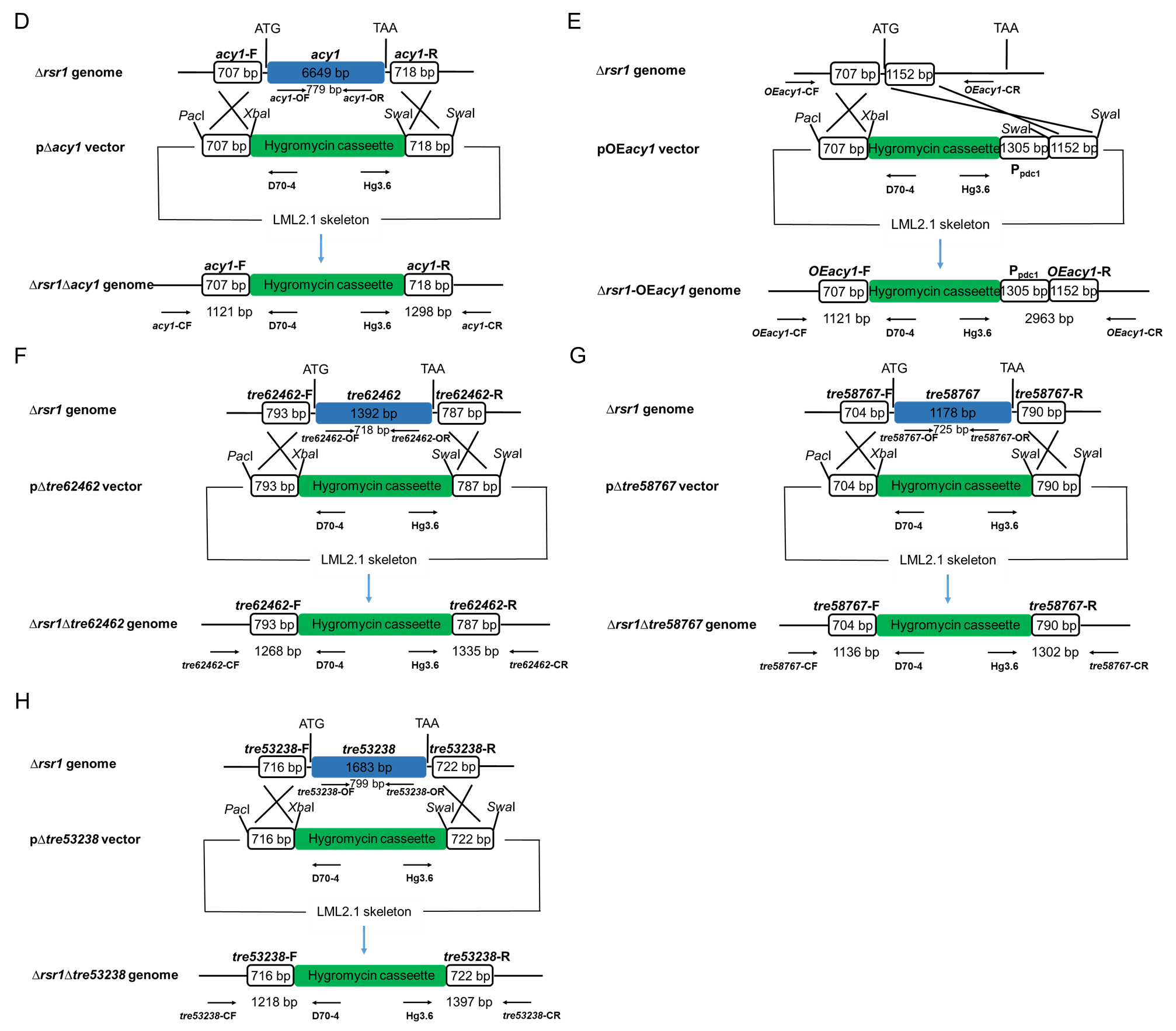


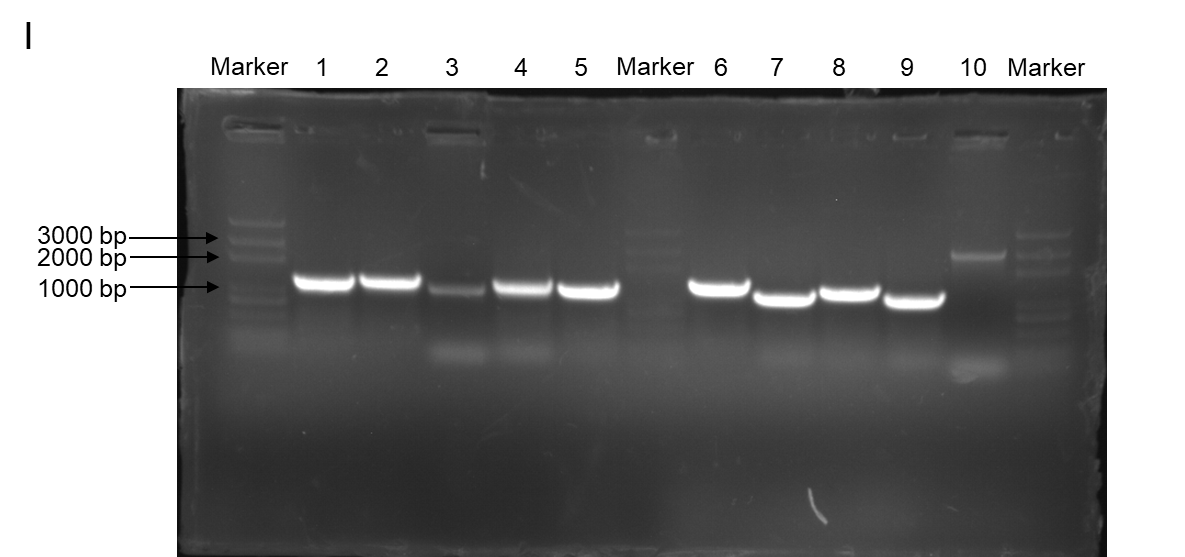


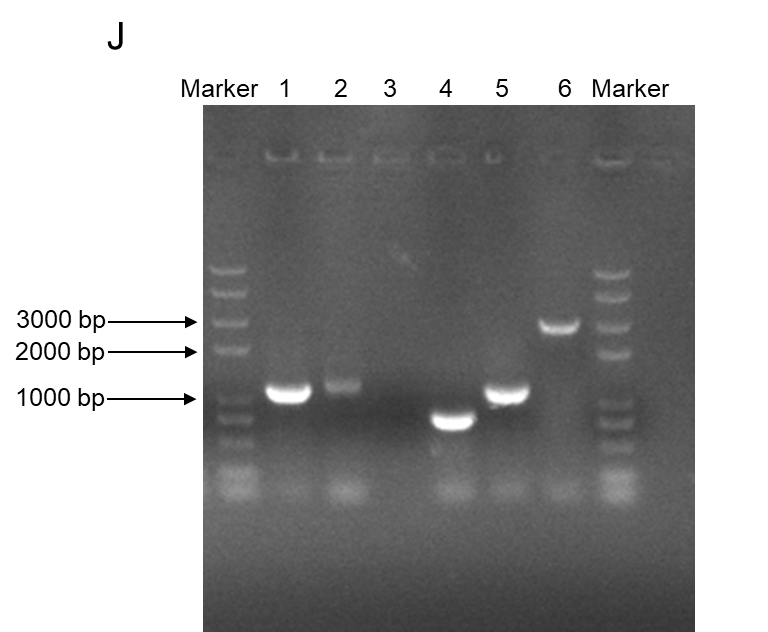


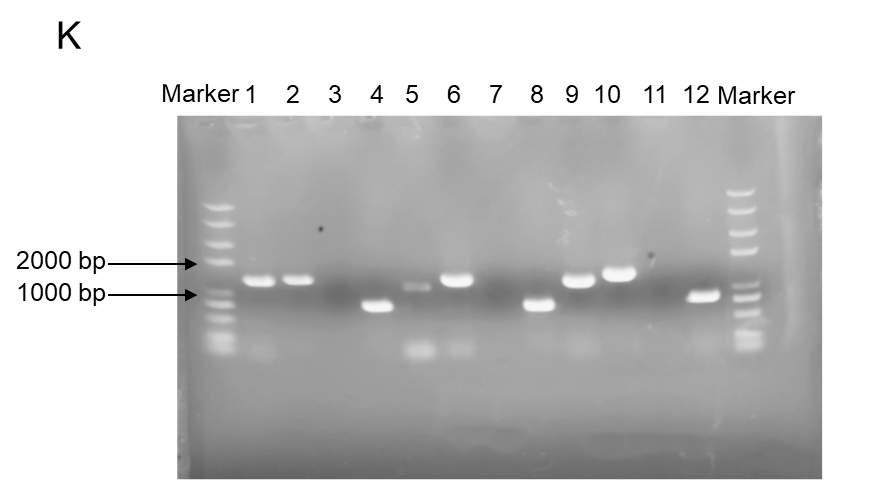


**Figure S3.** Construction and verification of deletion mutants. A. To construct an *rsr1* deletion mutant, the 758-bp upstream and 701-bp downstream fragments of *rsr1* were amplified from *T. reesei* QM6a genomic DNA were amplified by PCR. Then, upstream and downstream fragments were attached to the *Pac*I/*Xba*I and *Swa*I sites in an orderly manner, respectively, of linearized LML2.1 [1], to form the *rsr1* deletion plasmid (p∆*rsr1*). The deletion cassettes were transformed into QM6a strain by *Agrobacterium*-mediated transformation. The primers *rsr1*-CF/D70-4, Hg3.6/*rsr1*-CR and *rsr1*-OF/ *rsr1*-OR were used to verify the putative *rsr1* disruption mutants (Δ*rsr1*) by diagnostic PCR using with DNA sequencing. Hyg casseette were used as screening markers. B. To construct an RSR1 complementation strain, the 1.5-kb promoter fragment, gene coding sequence fragment, and 0.5-kb terminator fragment together were obtained from the *T. reesei genome* QM6a, which constituted 3245-bp and inserted into *Swa*I-linearized LML2.1 to turn into the re-complementation vector pR*rsr1* [2]. The re-complementation cassettes were transformed into Δ*rsr1* strain by *Agrobacterium*-mediated transformation. Re-complementation strains (RC-*rsr1*) are screened by PCR using the primers rc-*rsr1*-CF and rc-*rsr1*-CR and DNA sequencing. The primers were used in this experiment in Table S3. C. Validated electrophoretic diagram to verify the knockout and complementation of *rsr1*. D. The skeleton schematic diagram to knockout *acy1* in the knockout strain Δ*rsr1*. E. The skeleton schematic diagram to overexpress *acy1* in the knockout strain Δ*rsr1*. F. The skeleton schematic diagram to knockout *tre62462* in the knockout strain Δ*rsr1*. G. The skeleton schematic diagram to knockout *tre58767* in the knockout strain Δ*rsr1*. H. The skeleton schematic diagram to knockout *tre53238* in the knockout strain Δ*rsr1*. I. Validated electrophoretic diagram to verify the deletion of *tre62462*, *tre58767*, *tre53238*, *acy1* in the knockout strain Δ*rsr1*, and the overexpression of *acy1* in the knockout strain Δ*rsr1*. 1: ∆*rsr1*∆*tre62462*-F; 2: ∆*rsr1*∆*tre62462*-R; 3: ∆*rsr1*∆*tre58767*-F; 4: ∆*rsr1*∆*tre58767*-R; 5: ∆*rsr1*∆*tre53238*-F; 6: ∆*rsr1*∆*tre53238*-R; 7: ∆*rsr1*∆*acy1*-F; 8: ∆*rsr1*∆*acy1*-R; 9: ∆*rsr1*-OE*acy1*-F; 10: ∆*rsr1*-OE*acy1*-R. J. Validated electrophoretic diagram to verify the deletion of *acy1* in the original strain QM6a, and the overexpression of *acy1* in the original strain QM6a. 1: ∆*acy1*-F; 2: ∆*acy1*-R; 3: ∆*acy1*-O; 4: QM6a-O; 5: QM6a-OE*acy1*-F; 6: QM6a-OE*acy1*-R. K. Validated electrophoretic diagram to verify the deletion of *tre62462*, *tre58767*, and *tre53238* in the original strain QM6a. 1: ∆*tre62462*-F; 2: ∆*tre62462*-R; 3: ∆*tre62462*-O; 4: QM6a-O; 5: ∆*tre58767*-F; 6: ∆*tre58767*-R; 7: ∆*tre58767*-O; 8: QM6a-O; 9: ∆*tre53238*-F; 10: ∆*tre53238*-R; 11: ∆*tre53238*-O; 12: QM6a-O.

**References**

1. Zhang GX, Liu P, Wei W, Wang XD, Wei DZ, Wang W. A light-switchable bidirectional expression system in filamentous fungus *Trichoderma reesei*. J Biotechnol. 2016;240:85-93.
2. Chen YM, Shen YL, Wang W, Wei DZ. Mn^2+^ modulates the expression of cellulase genes in *Trichoderma reesei* Rut-C30 via calcium signaling. Biotechnol Biofuels. 2018;11:54.
