# Supplementary material for: The Ras small GTPase RSR1 regulates cellulase production in Trichoderma reesei
Source: Biotechnol Biofuels Bioprod. 2023 May 23;16:87. doi: 10.1186/s13068-023-02341-z (PMC10204303; doi:10.1186/s13068-023-02341-z)
Supplement: Supplementary file 6 — Additional file 6: Table S3. Comparison of cellulase and hemicellulase genes expression levels in Δrsr1 and QM6a strains [file 13068_2023_2341_MOESM6_ESM.docx]

**Table S3** Comparison of cellulase and hemicellulase genes expression levels in Δ*rsr1* and QM6a strains

| **Gene ID** | **Annotations** | **Log_2_fc** | **Up/Down** |
| --- | --- | --- | --- |
| **Cellulose degradation related genes** | | | |
| 123989 | cellobiohydeolase CBH1/CEL7A | 1.816920056 | up |
| 72567 | cellobiohydeolase CBH2/CEL6A | 1.881372012 | up |
| 122081 | endoglucanase EGL1/CEL7B | 2.905629352 | up |
| 120312 | endoglucanase EGL2/CEL5A | 1.86581686 | up |
| 123232 | endoglucanase EGL3/CEL12A | 2.257029922 | up |
| 73643 | endoglucanase EGL4/CEL61A | 0.747422664 | up |
| 49976 | endoglucanase EGL5/CEL45A | 2.033440952 | up |
| 120749 | beta-glucosidase BGL2/CEL1A | 1.162754232 | up |
| 69944 | alpha-glucosidase | 1.305172886 | up |
| 82227 | beta-glucosidase CEL3C | 1.159402749 | up |
| 73638 | cellulose-binding protein CIP1 | 1.875562476 | up |
| 123940 | cellulose-binding protein CIP2 | 0.750868159 | up |
| 123992 | Swollenin | 2.402427495 | up |
| 76672 | cel3a | 2.165727742 | up |
| 22197 | putative beta-glucosidase CELB | 0.925803564 | up |
| 46816 | beta-glucosidase CEL3D | 1.651359285 | up |
| 60085 | alpha-glucosidase | 0.348458539 | NS |
| 121351 | alpha-glucosidase GLS2 | 0.420169711 | up |
| 22072 | alpha-glucosidase | 0.4809757 | up |
| 50215 | beta-glycosidase | 1.188496978 | up |
| 108672 | alpha-1/3-glucanase | 0.35202121 | NS |
| **Hemicellulose degradation related genes** | | | |
| 74223 | xylanase XYN1 | 1.899379515 | NS |
| 123818 | xylanase XYN2 | 1.856425125 | up |
| 120229 | xylanase XYN3 | 1.684103158 | up |
| 111849 | xylanase XYN4 | 2.300072798 | up |
| 121127 | beta-xylosidase BXL1 | 1.923932009 | up |
| 73632 | acetyl xylan esterase AXE1 | 1.329936906 | NS |
| 56996 | mannan endo-1,4-beta-mannosidase MAN1 | 1.929082729 | up |
| 55999 | alpha-galactosidase | 1.294355832 | up |
| 72526 | alpha-glucuronidase GLR1 | 1.573485135 | up |
| 72704 | alpha-galactosidase AGL3 | 0.772588477 | NS |
| 55802 | alpha-1,6-mannanase | 1.530218994 | up |
| 67844 | alpha-1,6-mannanase | 0.196001042 | NS |
| 27259 | alpha-galactosidase | 1.351517056 | NS |

NS represented not significant, *P* adjust > 0.05
