# Supplementary material for: The Ras small GTPase RSR1 regulates cellulase production in Trichoderma reesei
Source: Biotechnol Biofuels Bioprod. 2023 May 23;16:87. doi: 10.1186/s13068-023-02341-z (PMC10204303; doi:10.1186/s13068-023-02341-z)
Supplement: Supplementary file 7 — Additional file 7: Table S4. Log2 fold change (Log2fc) of the top 10 genes differentially expressed in Δrsr1 and QM6a strains [file 13068_2023_2341_MOESM7_ESM.docx]

**Table S4** Log_2_ fold change (Log_2_fc) of the top 10 genes differentially expressed in Δ*rsr1* and QM6a strains

| **Condition** | **Gene ID** | **Description** | **Log_2_fc** |
| --- | --- | --- | --- |
| Up | 73536 | NADP-dependent glutamate dehydrogenase | 3.297408536 |
|  | 110035 | Predicted protein | 3.214404155 |
|  | 66999 | Acyl-CoA ligase | 3.073353841 |
|  | 104227 | Predicted protein | 3.045594307 |
|  | 56448 | Endochitinase 1 | 3.03070413 |
|  | 66228 | Xanthine/uracil permease | 3.013127355 |
|  | 106936 | Ferric/cupric reductase transmembrane component | 2.994913826 |
|  | 122081 | Endoglucanase EG-1 | 2.905629352 |
|  | 58450 | Exo-1,4-beta-xylosidase bxlB | 2.83762592 |
|  | 108143 | Predicted protein | 2.71554388 |
| Down | 76880 | RAS-GTPase RSR1 | -3.014773632 |
|  | 5084 | Conidiation-specific protein | -2.67948964 |
|  | 106131 | Predicted protein | -2.586010834 |
|  | 46285 | Heat shock protein | -2.536827453 |
|  | 107853 | Predicted protein | -1.696710744 |
|  | 62100 | Heat shock protein | -1.686440752 |
|  | 30578 | DNA glycosylase | -1.587721205 |
|  | 109284 | Predicted protein | -1.556573399 |
|  | 67738 | Longiborneol synthase | -1.552206383 |
|  | 121877 | Epoxide hydrolase | -1.535417646 |
