# Supplementary material for: The Ras small GTPase RSR1 regulates cellulase production in Trichoderma reesei
Source: Biotechnol Biofuels Bioprod. 2023 May 23;16:87. doi: 10.1186/s13068-023-02341-z (PMC10204303; doi:10.1186/s13068-023-02341-z)
Supplement: Supplementary file 8 — Additional file 8: Table S5. Log2fold change (Log2fc) of characterized transcriptional factors involved in the regulation of lignocellulase genes. NS represented not significant, P adjust > 0.05 [file 13068_2023_2341_MOESM8_ESM.docx]

**Table S5** Log_2_fold change (Log_2_fc) of characterized transcriptional factors involved in the regulation of lignocellulase genes. NS represented not significant, *P* adjust > 0.05

|  |  |  |  |  |  |
| --- | --- | --- | --- | --- | --- |
| Gene ID | Transcription factor genes | Log_2_fc | Up/Down | Positive/ Negative-acting | *P* adjust |
| 77513 | ACE3 | 1.447031547 | up | Positive | 2.07207E-10 |
| 122208 | XYR1 | 0.766736078 | up | Positive | 0.006403219 |
| 52368 | BglR | 0.372849514 | up | Positive | 0.003944617 |
| 76817 | AreA | 0.352378556 | up | Positive | 0.049799388 |
| 75418 | ACE1 | 0.139989101 | NS | Negative | 0.62334033 |
| 78445 | ACE2 | 0.310510146 | NS | Positive | 0.337334936 |
| 120117 | Cre1 | 0.363600401 | NS | Negative | 0.507067636 |
| 120698 | PacC | 0.052317368 | NS | Negative | 0.838577631 |
| 27600 | CLR-1 | 0.240890912 | NS | Positive | 0.297238206 |
| 26163 | CLR-2 | 0.514524544 | NS | Positive | 0.347092442 |
| 124286 | HAP2 | -0.173293933 | NS | Positive | 0.718092752 |
| 121080 | HAP3 | -0.136698276 | NS | Positive | 0.535440375 |
|  |  |  |  |  |  |
