# Supplementary material for: The Ras small GTPase RSR1 regulates cellulase production in Trichoderma reesei
Source: Biotechnol Biofuels Bioprod. 2023 May 23;16:87. doi: 10.1186/s13068-023-02341-z (PMC10204303; doi:10.1186/s13068-023-02341-z)
Supplement: Supplementary file 10 — Additional file 10: Table S7. The changes of 58 GPCR genes [1] in Δrsr1 and QM6a strains were sorted out, NS represented not significant, P adjust > 0.05 [file 13068_2023_2341_MOESM10_ESM.docx]

**Table S7** The changes of 58 GPCR genes [1] in Δ*rsr1* and QM6a strains were sorted out, NS represented not significant, *P* adjust > 0.05

|  |  |  |  |  |
| --- | --- | --- | --- | --- |
| **Gene ID** | **GPCR class** | **Log_2_fc** | ***P* adjust** | **Up/Down** |
| 64018 | I (pheromone receptors) | -0.252899309 | 0.7438859 | NS |
| 57526 | II (pheromone receptors) | -0.042231759 | 0.982659543 | NS |
| 59778 | III (related to A. nidulans GprC, GprD, and GprE) | -0.077483304 | 0.935527553 | NS |
| 80125 | IV (nitrogen sensors) | -0.169455474 | 0.718811976 | NS |
| 4508 | IV (nitrogen sensors) | -0.221070411 | 0.767128277 | NS |
| 72004 | V (cAMP receptor-like) | × | × | × |
| 72627 | V (cAMP receptor-like) | × | × | × |
| 123806 | V (cAMP receptor-like) | -0.552815104 | 0.410763127 | NS |
| 72605 | V (cAMP receptor-like) | 0 | 1 | NS |
| 63981 | VI (GPCRs containing RGS domain) | -0.577652943 | 0.147798311 | NS |
| 81383 | VI (GPCRs containing RGS domain) | 0.510202898 | 0.556903523 | NS |
| 37525 | VI (GPCRs containing RGS domain) | -0.24588706 | 0.673242173 | NS |
| 53238 | VII (related to rat growth hormone releasing  factor) | 1.158695926 | 0.000705892 | up |
| 119819 | VIII (related to human steroid receptor mPR) | 0.250059266 | 0.574577552 | NS |
| 68212 | VIII (related to human steroid receptor mPR) | 0.032750492 | 0.980117192 | NS |
| 70139 | VIII (related to human steroid receptor mPR) | 0.105253311 | 0.902638001 | NS |
| 82246 | VIII (related to human steroid receptor mPR) | 0.53716952 | 0.648157526 | NS |
| 56426 | VIII (related to human steroid receptor mPR) | -0.039062805 | 0.942020637 | NS |
| 5979 | X (similar to PTM1) | -0.020302691 | 0.95717307 | NS |
| 107503 | XI (similar to GPCR89) | 0.287418518 | 0.294899645 | NS |
| 55374 | XII (family C-like GPCRs) | -0.386116606 | 0.596577397 | NS |
| 120238 | XIII (related to GPR11 of P. sojae) | -0.291062242 | 0.235576323 | NS |
| 27948 | XIII (related to GPR11 of P. sojae) | -0.156515384 | 0.663239668 | NS |
| 69500 | PTH11-like GPCRs | 0.195268194 | 0.836631719 | NS |
| 122795 | PTH11-like GPCRs | 0.2599486 | 0.805797335 | NS |
| 45573 | PTH11-like GPCRs | 0.94477341 | 2.91674E-05 | up |
| 5647 | PTH11-like GPCRs | -0.021623127 | 0.990037173 | NS |
| 27992 | PTH11-like GPCRs | -0.047318776 | 0.941608597 | NS |
| 27983 | PTH11-like GPCRs | 0.635958981 | 0.787756418 | NS |
| 62462 | PTH11-like GPCRs | 1.26322777 | 1.60043E-10 | up |
| 53452 | PTH11-like GPCRs | 0 | 1 | NS |
| 66786 | PTH11-like GPCRs | -0.998514391 | 0.224691109 | NS |
| 106082 | PTH11-like GPCRs | -0.360971555 | 0.716991573 | NS |
| 55561 | PTH11-like GPCRs | 0.129177877 | 0.847598332 | NS |
| 67334 | PTH11-like GPCRs | 0.377283312 | 0.084506518 | NS |
| 61354 | PTH11-like GPCRs | 0.389427507 | 0.75995087 | NS |
| 122824 | PTH11-like GPCRs | 0.342924062 | 0.888381551 | NS |
| 40156 | PTH11-like GPCRs | 0.468845589 | 0.27954702 | NS |
| 70967 | PTH11-like GPCRs | 1.319688877 | 0.062600106 | NS |
| 58767 | PTH11-like GPCRs | 1.430158452 | 0.00070725 | up |
| 41260 | PTH11-like GPCRs | 1.229939063 | 1 | NS |
| 110339 | PTH11-like GPCRs | × | × | × |
| 110744 | PTH11-like GPCRs | -0.020963394 | 0.97467001 | NS |
| 111861 | PTH11-like GPCRs | -1.631968139 | 1 | NS |
| 69904 | PTH11-like GPCRs | 1.336102687 | 0.196594265 | NS |
| 41425 | PTH11-like GPCRs | 0.828526037 | 0.666144008 | NS |
| 105224 | PTH11-like GPCRs | 1.364077999 | 0.469005931 | NS |
| 57101 | PTH11-like GPCRs | 2.813531813 | 1 | NS |
| 124113 | PTH11-like GPCRs | 0.353357526 | 0.574883684 | NS |
| 66673 | PTH11-like GPCRs | 0 | 1 | NS |
| 76763 | PTH11-like GPCRs | 0.966328517 | 0.237928414 | NS |
| 39587 | PTH11-like GPCRs | -1.715666194 | 1 | NS |
| 121990 | PTH11-like GPCRs | 0.465942649 | 0.623852815 | NS |
| 109146 | PTH11-like GPCRs | -1.225427296 | 0.387927769 | NS |
| 78499 | PTH11-like GPCRs | -0.107326368 | 0.884166399 | NS |
| 107042 | PTH11-like GPCRs | 1.299309153 | 0.068748138 | NS |
| 103694 | PTH11-like GPCRs | -0.087111308 | 0.859595037 | NS |
| 82041 | PTH11-like GPCRs | 0.117733532 | 0.919728219 | NS |

× represents not detected.

**References**

1. Gruber S, Omann M, Zeilinger S. Comparative analysis of the repertoire of G protein-coupled receptors of three species of the fungal genus *Trichoderma*. BMC Microbiol. 2013;13:108.
