# Supplementary material for: The Ras small GTPase RSR1 regulates cellulase production in Trichoderma reesei
Source: Biotechnol Biofuels Bioprod. 2023 May 23;16:87. doi: 10.1186/s13068-023-02341-z (PMC10204303; doi:10.1186/s13068-023-02341-z)
Supplement: Supplementary file 11 — Additional file 11: Table S8 Primers used in this study [file 13068_2023_2341_MOESM11_ESM.docx]

**Table S8** Primers used in this study.

| Primer | oligos Sequences (5’ to 3’) |
| --- | --- |
| **Construction of the gene deletion vector** | |
| *TrRas1*-D1 | ATTACGAATTCTTAATTAATGAACCAACTGGAACCTCACTA |
| *TrRas1*-D2 | CATTATACGAAGTTATTCTAGACTCAGCCAATGAAGGACAAGG |
| *TrRas1*-D3 | ACTAGTGAGCTCATTTAGGCGACATTCATGGTGGAA |
| *TrRas1*-D4 | AGTGCCAAGCTTATTTGTTAGCAAGGATCAAGGCAGAG |
| *TrRas2*-D1 | ATTACGAATTCTTAATTAAATGGCATGGCATGGCATTG |
| *TrRas2*-D2 | CATTATACGAAGTTATTCTAGAGCAGAAGCAGAAGCAAGAGG |
| *TrRas2*-D3 | ACTAGTGAGCTCATTTCTCATTCGTTGCCGCTTCC |
| *TrRas2*-D4 | AGTGCCAAGCTTATTTGCGACCATCCATTCCATATCTG |
| *rsr1*-D1 | ATTACGAATTCTTAATTAAAGAGAGGAAAGGCAACAAGAGA |
| *rsr1*-D2 | CATTATACGAAGTTATTCTAGAGCAGGTCAGAGGACAATGAAG |
| *rsr1*-D3 | ACTAGTGAGCTCATTTTCTTTCCTGTGCTCCCTCTTC |
| *rsr1*-D4 | AGTGCCAAGCTTATTTTCTTCGTCTCTTCTACCGTTGT |
| *tre107035*-D1 | ATTACGAATTCTTAATTAAGGTGAGTGGATTCGCAAAGG |
| *tre107035*-D2 | CATTATACGAAGTTATTCTAGACGGAGGATGAGGAGAGTTGG |
| *tre107035*-D3 | ACTAGTGAGCTCATTTCCGAGACAGACGAGATCAA |
| *tre107035*-D4 | AGTGCCAAGCTTATTTGCAGGCAGTAATGGAATGG |
| *tre66480*-D1 | ATTACGAATTCTTAATTAACGCCATCAACAAGACTCTGAAT |
| *tre66480*-D2 | CATTATACGAAGTTATTCTAGAGAGAATGGGAAATGAGGCTGAG |
| *tre66480*-D3 | ACTAGTGAGCTCATTTTCAGAGACCGAGCCCAAGA |
| *tre66480*-D4 | AGTGCCAAGCTTATTTAGTTGAGATTGACGCCAAGATC |
| *tre34726*-D1 | ATTACGAATTCTTAATTAACTCCTCACCTCACCACCATC |
| *tre34726*-D2 | CATTATACGAAGTTATTCTAGAGCCAATGTCTCTGACTCAAACT |
| *tre34726*-D3 | ACTAGTGAGCTCATTTCAAGCCGCCTACTCATTCAAG |
| *tre34726*-D4 | AGTGCCAAGCTTATTTCCTCCACACGACACGCATA |
| *tre107369*-D1 | ATTACGAATTCTTAATTAAACCATCAGTCTGTGCCATCC |
| *tre107369*-D2 | CATTATACGAAGTTATTCTAGATCAGAAGGCGAGTGCTGTC |
| *tre107369*-D3 | ACTAGTGAGCTCATTTGCCGCAGAGGACACGATAT |
| *tre107369*-D4 | AGTGCCAAGCTTATTTCAGACACAGCCACATCACAC |
| *tre67275*-D1 | ATTACGAATTCTTAATTAATCGTGTAAGTCCACTCTCAGTC |
| *tre67275*-D2 | CATTATACGAAGTTATTCTAGAGAGCAGGAGTAGTCGTCTTCTT |
| *tre67275*-D3 | ACTAGTGAGCTCATTTAACCTCACATACCTGCCTCTAC |
| *tre67275*-D4 | AGTGCCAAGCTTATTTGGCTACTCGTTAACATCACTCC |
| *tre70548*-D1 | ATTACGAATTCTTAATTAAGCTAGAGGCAAGGTAGCAGAA |
| *tre70548*-D2 | CATTATACGAAGTTATTCTAGAGGGAGAATGGAGATGGAGAGG |
| *tre70548*-D3 | ACTAGTGAGCTCATTTTGAACAAGCGGCGTATATTGG |
| *tre70548*-D4 | AGTGCCAAGCTTATTTGGACCTTCGTTCTACTCTTCCA |
| *tre61408*-D1 | ATTACGAATTCTTAATTAATGACGCTCTTCGCCTTCTC |
| *tre61408*-D2 | CATTATACGAAGTTATTCTAGAAGAGGTTGACGCTGTGGAG |
| *tre61408*-D3 | ACTAGTGAGCTCATTTGCCTGTAACGATGCTTGCTT |
| *tre61408*-D4 | AGTGCCAAGCTTATTTGGTTCCAGTGGTGGTGGTT |
| *tre81785*-D1 | ATTACGAATTCTTAATTAAGGCAGCATCGTCGGTATTG |
| *tre81785*-D2 | CATTATACGAAGTTATTCTAGATGGAGGAGGTCAGGTGTGA |
| *tre81785*-D3 | ACTAGTGAGCTCATTTACCAACGACAAGTCTGTACCA |
| *tre81785*-D4 | AGTGCCAAGCTTATTTCCCATTCCTCTAGCCCATCC |
| *acy1*-D1  *acy1*-D2  *acy1*-D3  *acy1*-D4 | ATTACGAATTCTTAATTAACCACCAGACAAGACAAGACAAG  CATTATACGAAGTTATTCTAGACGAGTGGTATTGGTCGAGGT  ACTAGTGAGCTCATTTCGGAGTTGAGAAGAGGAGGTT  AGTGCCAAGCTTATTTAAAGGTTCGCCAGTTTGTTGA |
| *tre62462*-D1 | ATTACGAATTCTTAATTAACTCGCCGTCTGAGAAGGATAT |
| *tre62462*-D2 | CATTATACGAAGTTATTCTAGAAAGAGCACATACAGCACATGAG |
| *tre62462*-D3 | ACTAGTGAGCTCATTTACGGCTGCATCGTTGGAT |
| *tre62462*-D4 | AGTGCCAAGCTTATTTCGACCTTGCCTTCACAATCATA |
| *tre58767*-D1 | ATTACGAATTCTTAATTAATTCACTGCGGCACACGAA |
| *tre58767*-D2 | CATTATACGAAGTTATTCTAGATACGGAATAGTCTGCGAGAAGT |
| *tre58767*-D3 | ACTAGTGAGCTCATTTCGCAGCTCAACAGCATCAG |
| *tre58767*-D4 | AGTGCCAAGCTTATTTCTAGGGAACGCCTCCAAGAA |
| *tre53238*-D1 | ATTACGAATTCTTAATTAATCGCAACCTCAGAAGCTCC |
| *tre53238*-D2 | CATTATACGAAGTTATTCTAGACCCGAGATGTACGAGTGATGA |
| *tre53238*-D3 | ACTAGTGAGCTCATTTGATGCCGCGCTTTACTTCG |
| *tre53238*-D4 | AGTGCCAAGCTTATTTCCCGTCCTCATAGCACCTTT |
| **Verification of the gene deletion mutants** | |
| *TrRas1*-CF | GCCGCTTCTTCCCACTTTGTAC |
| *TrRas1*-CR | AGTCAGCATAGGCGTTCTGGAG |
| *TrRas1*-OF | AGAGTACAAGCTCGTCGTCGTT |
| *TrRas1*-OR | CTCCTCGCCCTCAATGTCCAT |
| D70-4 | TCGGACTTGCGGAGGATGTTGTAT |
| Hg3.6 | TGCCTAGTGAATGCTCCGTAACA |
| *TrRas2*-CF | CCGACCTACGAGTACAGCAAGT |
| *TrRas2*-CR | AGCCTCTAGCATTCAGCATCCT |
| *TrRas2*-OF | ACTTCGTCGAGACGGTAAGCAT |
| *TrRas2*-OR | ATTGATGCAGTTCTTGGCCGAG |
| *rsr1*-CF | GAAAGAAAGGCGACGGCAAGG |
| *rsr1*-CR | CCAAAGCGATGCGGAAAGAACT |
| *rsr1*-OF | TTACTTAGGTGGCGTCGGCAA |
| *rsr1*-OR | TGGTTCTGGCACTGGCTTCATA |
| *tre107035*-CF | CCTTGACGCTCCTTGTCCTCT |
| *tre107035*-CR | CGGCGACATCCACATCCTCT |
| *tre107035*-OF | TGCCCATCTCCATCACCATCTG |
| *tre107035*-OR | TCACCAACACCGCAACTTCTTC |
| *tre66480*-CF | CCCGAGCGTCCCTTTATACAGA |
| *tre66480*-CR | GCTGACAAGGGAGCAGTGGTA |
| *tre66480*-OF | CCTTCTCCGCTCGCCATGAA |
| *tre66480*-OR | TGCTAGTTTCTTGCCCTCCTCG |
| *tre34726*-CF | TTCTCTTCCCTCCTCCTCTCCC |
| *tre34726*-CR | ACGACCAGCCCTACCTCTTCA |
| *tre34726*-OF | GCTGACAGTTGTCGCCATTCC |
| *tre34726*-OR | CCATCTCGCCAATCTCATCTGC |
| *tre107369*-CF | GCAACCGTCAAGCGTCATTCT |
| *tre107369*-CR | AATGACGGCGGGAGGTTCTG |
| *tre107369*-OF | GACTGGTTGCCGAGATCACATC |
| *tre107369*-OR | ACAATGTCCATGCTCGCTATGC |
| *tre67275*-CF | TCGAGTCGAGTTCAGCCAAGTC |
| *tre67275*-CR | ACTTCCAACCGCCTGTTCACA |
| *tre67275*-OF | CCTGGTGTCTGAGCGTCTAAGT |
| *tre67275*-OR | GCCAACGGATGTAGCAGAGGA |
| *tre70548*-CF | TTGGATGTGAGGTGAGGTGAGG |
| *tre70548*-CR | GCCAACGACATCAGACCAATCA |
| *tre70548*-OF | GTTCCTTCAGTGCCGTCCTCT |
| *tre70548*-OR | CCCTCTTCGTCGTCCAGAAAGT |
| *tre61408*-CF | AGTACCAGGCACAGGCTCCTA |
| *tre61408*-CR | CTTCGGCATCACGGAATTCACA |
| *tre61408*-OF | AGAACCGCATGGCTCTGGAAG |
| *tre61408*-OR | TCGATGATGGCTTGGACCTGAG |
| *tre81785*-CF | TGCCTGCCTGGATGTGACTG |
| *tre81785*-CR | ATCATCATGGCAGGCAACTTGG |
| *tre81785*-OF | CCAACCGAAGATGCGAACCG |
| *tre81785*-OR | TGGCAGCAGAGGCGTAGATG |
| *acy1*-CF | GCTGCTCTGGTCAAGCCTCT |
| *acy1*-CR | GGTCCACGGCTACGATGGTATA |
| *acy1*-OF | GGACTCCACGAGCAGCTCATAT |
| *acy1*-OR | TGCGAATGAAGTAGGGCGTCA |
| *tre62462*-CF | CCCTTTGGCGCAAACCAAGG |
| *tre62462*-CR | GACGAGTGGGAGAGCAAGGT |
| *tre62462*-OF | GACCCTCGCCCTTATCCTATGG |
| *tre62462*-OR | TGGCAAGCAGACGCAGATGA |
| *tre58767*-CF | GATGAGGCTATCCTCGGCAAGA |
| *tre58767*-CR | ACTGTGGCTGCTTCGGACTC |
| *tre58767*-OF | TGGGACGACATCTTCGCAGTAG |
| *tre58767*-OR | TGGTTCGGGTACTTGACAGTGT |
| *tre53238*-CF | AAGCATTGTGACGACGCATGAG |
| *tre53238*-CR | ACACCCATCATTTGCGAAGTCG |
| *tre53238*-OF | ATCGCAACCGCCAGTGTCA |
| *tre53238*-OR | GGAAGACGAAGACGACGGAGAA |
| **Construction of the *rsr1* gene re-complementation vector** | |
| rc-*rsr1*-1 | ACTAGTGAGCTCATTTTGTCCTCCTGGCGCAGC |
| rc-*rsr1*-2 | CAAGATGGTCACGGCTGCTG |
| rc-*rsr1*-3 | GCCGTGACCATCTTGTGC |
| rc-*rsr1*-4 | AGTGCCAAGCTTATTTAGAAAGGAAATAAAATAAAGGTAAGCAA |
| **Verification of the *rsr1* gene re-complementation mutants** | |
| rc-*rsr1*-CF | GTCCAGCATCCGTTTCGTCAAA |
| rc-*rsr1*-CR | GGCAAAGAAAGGTGCAATGGGA |
| rc-*rsr1*-OF | TTACTTAGGTGGCGTCGGCAA |
| rc-*rsr1*-OR | TGGTTCTGGCACTGGCTTCATA |
| **Construction of the *acy1* gene overexpression vector** | |
| *acy1*-OE-D1 | ATTACGAATTCTTAATTAACCACCAGACAAGACAAGACAAG |
| *acy1*-OE-D2 | CATTATACGAAGTTATTCTAGACGAGTGGTATTGGTCGAGGT |
| Rpdc-1 | ACTAGTGAGCTCATTTATGAAAGGAGGGAGCATTCTTCGA |
| Rpdc-2 | CATGATTGTGCTGTAGCTGCGC |
| *acy1*-OE-D3 | AGCTACAGCACAATCATGACGCCCTCGTCGTCCA |
| *acy1*-OE-D4 | AGTGCCAAGCTTATTTCGCAGGCTCCTGGGATCATC |
| **Verification of the *acy1* gene overexpression mutants** | |
| *acy1*-OE-CF | GCTGCTCTGGTCAAGCCTCT |
| *acy1*-OE-CR | GCCATCGTCCTTGTCCTTGTG |
| **Quantitative RT-PCR analysis** | |
| Q*sar1*-1 | TGGATCGTCAACTGGTTCTACGA |
| Q*sar1*-2 | GCATGTGTAGCAACGTGGTCTTT |
| Q*cbh1*-1 | CTCCATCTCCGAGGCTCTTACC |
| Q*cbh1*-2 | GCAAGTGCCGCCATATCTGTTAT |
| Q*cbh2*-1 | GCATATTACGCCTCTGAAGTTAGCA |
| Q*cbh2*-2 | GCATAGTTACCGCCATTCTTGTTG |
| Q*egl1*-1 | GCAGCCTCACCATGAACCAGTA |
| Q*egl1*-2 | CACCGTCAGAGTCCAGGAGATAC |
| Q*egl2*-1 | TGAACAAGTCCGTGGCTCCAT |
| Q*egl2*-2 | ACAATTCGTAGGTCCGCTCCAA |
| Q*xyn1*-1 | GGTTGGACGACTGGATCT |
| Q*xyn1*-2 | GGTTGTCCTCCATGATGTAG |
| Q*xyn2*-1 | CATCGTCGAGAACTTTGGCA |
| Q*xyn2*-2 | GCGTGCGGTAAATGTCGTAG |
| Q*cre1*-1 | CTCAAGGCGCGGCAACAAGG |
| Q*cre1*-2 | GAGTGCGGAGGCGAGACGTT |
| Q*acy1*-1 | CAGCGGACATGCGCCCTACT |
| Q*acy1*-2 | GGCGCCATCGTCCTTGTCCT |
| Q*pkac1*-1 | GCAGCAGCAGCAGCACCATC |
| Q*pkac1*-2 | GCTGTGGCCGTGGTGGATCT |
| Q*pkac2*-1 | TCTCGTCCGCCCTGCCAATG |
| Q*pkac2*-2 | TCGTGGCGCACATGGTCGAT |
| Q*pkar1*-1 | TGCACAGCGTCGTGGAGGAG |
| Q*pkar1*-2 | GTTGATGCGTCGCCGCCAAA |
|  |  |
