# Supplementary material for: The Ras small GTPase RSR1 regulates cellulase production in Trichoderma reesei
Source: Biotechnol Biofuels Bioprod. 2023 May 23;16:87. doi: 10.1186/s13068-023-02341-z (PMC10204303; doi:10.1186/s13068-023-02341-z)
Supplement: Supplementary file 12 — Additional file 12: Figure S4. The correlation between the biological replicates of each sample. The following heat map is used to show the correlation. Each value in the grid is the correlation coefficient between two samples. The larger the value, the greater the correlation between the two samples and the closer they are. These results indicate that the transcriptome data is more credible [file 13068_2023_2341_MOESM12_ESM.docx]

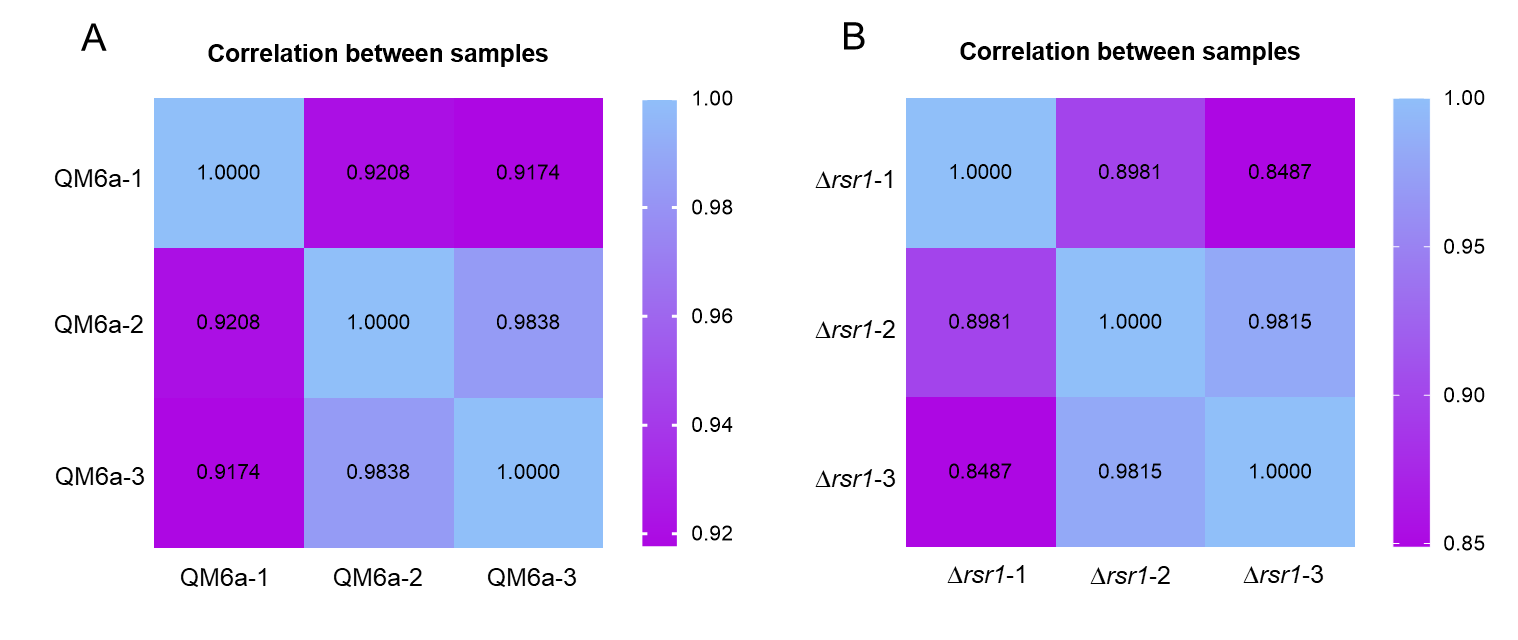


**Figure S4.** The correlation between the biological replicates of each sample. The following heat map is used to show the correlation. Each value in the grid is the correlation coefficient between two samples. The larger the value, the greater the correlation between the two samples and the closer they are. These results indicate that the transcriptome data is more credible.
